# Criteria for trauma team activation and staffing requirements for the management of patients with (suspected) multiple and/or severe injuries in the resuscitation room– a systematic review and clinical practice guideline update

**DOI:** 10.1007/s00068-025-02817-7

**Published:** 2025-03-18

**Authors:** Christian Alexander Kühne, Alina Weise, Nadja Könsgen, Uwe Schweigkofler, Arnold Kaltwasser, Sabrina Pelz, Tobias Becker, Christopher Spering, Frithjof Wagner, Dan Bieler

**Affiliations:** 1 Department for Trauma and Hand Surgery, Center for Geriatric Trauma, Schön Clinic Hamburg, 22081 Hamburg, Germany; 2 Institute for Research in Operative Medicine (IFOM), Witten/Herdecke University, Cologne, Germany; 3 Department of Trauma and Orthopaedic Surgery, BG Unfallklinik Frankfurt, Frankfurt am Main, Germany; 4 Kreiskliniken Reutlingen, Academy of the District Hospitals Reutlingen, Reutlingen, Germany; 5 Intensive Care Unit, University Hospital Tübingen, Tübingen, Germany; 6 Berufsgenossenschaftliche Unfallklinik Murnau, National Trauma Center, Education Center, Murnau, Germany; 7 Department of Trauma Surgery, Orthopaedics, University Medical Center, Goettingen, Germany; 8 Department Septische und Rekonstruktive Chirurgie, Berufsgenossenschaftliche Unfallklinik Murnau, Murnau, Germany; 9 Department of Orthopaedics and Trauma Surgery, Reconstructive Surgery, Hand Surgery, Plastic Surgery and Burn Medicine, German Armed Forces Central Hospital Koblenz, Koblenz, Germany; 10 Department of Orthopaedics and Trauma Surgery, Medical Faculty University Hospital Duesseldorf, Heinrich-Heine-University, Duesseldorf, Germany

**Keywords:** Trauma team activation, Trauma triage, Trauma room, Emergency room, Guideline, Severely injured, Polytrauma

## Abstract

**Purpose:**

Our aim was to update the evidence-based and consensus-based recommendations on criteria for trauma team activation (TTA) and staffing requirements for the management of patients with (suspected) multiple and/or severe injuries in the resuscitation room on the basis of available evidence. This guideline topic is part of the 2022 update of the German Guideline on the Treatment of Patients with Multiple and/or Severe Injuries.

**Methods:**

MEDLINE and Embase were systematically searched to August 2021. Further literature reports were obtained from clinical experts. Randomised controlled trials, prospective cohort studies, cross-sectional studies and comparative registry studies were included if they compared criteria for identifying severely injured patients requiring trauma team activation or different staffing components (e.g. team composition, training) for the management of patients with (suspected) multiple and/or severe injuries in the resuscitation room. We considered patient relevant outcomes such as mortality as well as prognostic accuracy outcomes. Risk of bias was assessed using NICE 2012 checklists. The evidence was synthesised narratively, and expert consensus was used to develop recommendations and determine their strength.

**Results:**

Twenty-one new studies were identified. Potential trauma team activation criteria included vital signs (e.g. systolic blood pressure), type and extent of injury (e.g. central gunshot wound), mechanism of injury (e.g. traffic accident), interventions (e.g. chest tube), specific criteria for geriatric patients, and combined criteria (N = 20). Staffing requirements for the resuscitation room included specific training for orthopaedic trainees (N = 1). Two recommendations were modified, and six additional recommendations were developed. All but two recommendations achieved strong consensus.

**Conclusion:**

The key recommendations address the following topics: inter-professional trauma teams in the resuscitation room; trauma team activation for geriatric patients; and trauma team activation criteria based on physiological, anatomical, interventional, and mechanism of injury parameters.

**Supplementary Information:**

The online version contains supplementary material available at 10.1007/s00068-025-02817-7.

## Introduction

In Germany, an estimated annual number of almost ten million people experience injuries, the majority of which occur at home or during leisure activities [1]. Severe injuries are among the leading causes of death in people under 45 years of age and are most commonly caused by road traffic accidents and falls. Every year, an estimated number of more than 32,000 severely injured patients are admitted to a hospital via the resuscitation room [2–4]. Today it is generally agreed that patient management in the resuscitation room and initial surgical care play a key role in patient outcome.

It is currently recommended that the decision to admit a patient with potentially severe injuries to the hospital resuscitation room be made on the basis of an evaluated set of criteria that is based on vital sign abnormalities, the assumed anatomical injury pattern, and the mechanism of injury [5]. This set of trauma team activation (TTA) criteria is included in the S3 Guideline on the Treatment of Patients with Multiple and/or Severe Injuries of 2016 [6].

The quality of activation criteria can be assessed on the basis of the rates of overtriage and undertriage, i.e. the percentage of patients who were admitted to the resuscitation room but did not need this type of care (overtriage) and the percentage of patients who were not initially admitted to the resuscitation room but urgently needed such care (undertriage). In the international literature, overtriage rates vary between 12% and 85% and undertriage rates between 0.4% and 21%. Publications from the United States show that, despite an overtriage rate of 72%, undertriage rates are still between 10% and 19% [7–9]. Studies from France present a different picture. These studies report an overtriage rate of 60% and an undertriage rate of merely 1% [10, 11].

Trauma team activation on the basis of mechanism of injury criteria has been associated with considerable overtriage in Germany in recent years [12–15]. An overview of criteria for TTA and staffing requirements is presented in the 2016 S3 Guideline of the German Association of the Scientific Medical Societies [6]. The objective of this review is to re-evaluate these criteria on the basis of international evidence and to provide validated criteria for TTA and staffing requirements which can be reliably assessed.

## Methods

This guideline topic is part of the 2022 update of the German Guideline on the Treatment of Patients with Multiple and/or Severe Injuries [16]. The guideline update is reported according to the RIGHT tool [17], the systematic review part according to the Preferred Reporting Items for Systematic Reviews and Meta-Analyses (PRISMA) 2020 reporting guideline [18]. The development and updating of recommendations followed the standard methodology set out in the guideline development handbook issued by the German Association of the Scientific Medical Societies (AWMF) [19]. All methods were defined a priori, following the methods report of the previous guideline version from July 2016 [20] with minor modifications, as detailed below.

### PICO questions and eligibility criteria

Population, intervention, comparison, and outcome (PICO) questions were retained from the previous guideline version. In addition, the participating professional societies involved in guideline development were asked to submit new PICO questions. The overarching PICO question for this topic area were:

*In adult patients (≥ 14 years) with known or suspected polytrauma and/or severe injuries*,

- *does trauma team activation according to specific criteria improve patient relevant outcomes or prognostic accuracy compared to any other criteria?*
- *do specific resuscitation room staffing requirements improve patient relevant outcomes or examination results compared to any other requirements?*

The full set of predefined PICO questions is listed in Table S1 (Online Resource 1). The study selection criteria in the PICO format are shown in Table 1.

**Table 1:** Predefined selection criteria

Population:	• Adult patients (≥ 14 years) with (suspected) polytrauma and/or severe injuries^a, b^ • Potential trauma team members, e.g. physicians, nurses
Intervention /comparison:	• Prognostic criteria identifying patients with severe injuries or need for emergency interventions in the context of trauma team activation / trauma triage • Staffing requirements for the management of patients in the resuscitation room
Outcomes:	• Any patient-relevant clinical outcomes, such as mortality • Prognostic accuracy • Examination results
Study type:	• Comparative, prospective studies (randomised controlled trials, cohort studies, cross-sectional studies) • Comparative registry^c^ data (incl. case-control studies) • Systematic reviews based on the above primary study types
Language:	English or German
Other inclusion criteria:	• Full text of study published and accessible • Study matches predefined PICO question
Exclusion criteria:	• Multiple publications of the same study without additional information • Study already included in previous guideline version

^a^ For PICOs on trauma team activation, the population had to be trauma patients. For PICOs on staffing requirements, the population had to be medical staff who treat patients who were admitted to the resuscitation room

^b^ For new PICO questions, indirect evidence from other populations was eligible for inclusion if direct evidence was unavailable

^c^ Using the Agency for Healthcare Research and Quality (AHRQ) definition of registries [21]

### Literature search

An information specialist systematically searched for literature in MEDLINE (Ovid) and Embase (Elsevier). The search strategy described in the 2011 Guideline was modified. It contained index (MeSH/Emtree) and free text terms for the population and intervention. All searches were completed on 27 August 2021. No start date was applied. Table S2 (Online Resource 1) provides details for all searches. Clinical experts were asked to submit additional relevant references.

### Study selection

Study selection was performed independently by two reviewers in a two-step process using the predefined eligibility criteria: (1) title/abstract screening of all references retrieved from database searches using Rayyan software [22] and (2) full-text screening of all articles deemed potentially relevant by at least one reviewer at the title/abstract level in Endnote (Endnote, Version: 20 [Software], Clarivate, Boston, Massachusetts, USA. https://endnote.com/). Disagreements were resolved through consensus or by consulting a third reviewer. The reasons for full-text exclusion were recorded (Table S3, Online Resource 1).

### Assessment of risk of bias and level of evidence

Two reviewers sequentially assessed the risk of bias of included studies at study level using the relevant checklists from the NICE guidelines manual 2012 [23] and assigned each study an initial level of evidence (LoE) using the Oxford Centre for Evidence-based Medicine Levels of Evidence (2009) [24]. The risk of bias for prognostic studies was not assessed since no relevant risk-of-bias tool for prognostic studies had been predefined in the guideline methods. For studies with baseline imbalance and unadjusted analyses, post-hoc secondary analyses, indirectness of the study population, or low power or imprecision of the effect estimate, the LoE was downgraded and marked with an arrow (↓). Any disagreements were resolved through consensus or by consulting a third reviewer.

### Data extraction and data items

Data were extracted into a standardised data table by one reviewer and checked by another. A predefined data set was collected for each study, consisting of study characteristics (study type, aims, setting), patient selection criteria and baseline characteristics (age, gender, injury scores, other relevant variables), intervention and control group treatments or (potential) trauma team activation criteria that were compared, patient flow (number of patients included and analysed), matching/adjusting variables, and data on outcomes for any time point reported.

### Outcome measures

Outcomes were extracted as reported in the study publications. For prospective cohort studies and registry data, preference was given to data obtained after propensity-score matching or statistical adjustment for risk-modulating variables over unadjusted data.

### Synthesis of studies

Studies were grouped by PICO questions. An interdisciplinary expert group used their clinical experience to synthesise studies narratively by balancing beneficial and adverse effects extracted from the available evidence. Clinical heterogeneity was explored by comparing inclusion criteria and patient characteristics at baseline.

### Development and updating of recommendations

For each PICO question, the following updating options were available: (1) the recommendation of the preceding version remains valid and requires no changes (“confirmed”); (2) the recommendation requires modification (“modified”); (3) the recommendation is no longer valid or required and is deleted; (4) a new recommendation needs to be developed (“new”). An interdisciplinary expert group of clinicians and nurses with expertise in the management of severe trauma and acute care reviewed the body of evidence, drafted recommendations based on the homogeneity of clinical characteristics and outcomes, the balance between benefits and harms, as well as their clinical expertise, and proposed grades of recommendation (Table 2). In the absence of eligible evidence, good practice recommendations were made based on clinical experience, data from studies with a low level of evidence, and expert consensus in cases where the Guideline Group felt a statement was required due to the importance of the topic. These were not graded, and instead labelled as good (clinical) practice points (GPP). For GPPs, the strength of a recommendation is presented in the wording shown in Table 2.

**Table 2:** Grading of recommendations

Symbol	Grade of recommendation	Description	Wording (examples)
⇑⇑	A	Strong recommendation	“use…”, “do not use…”
⇑	B	Recommendation	“should use…”, “should not use…”
⇔	0	Open recommendation	“consider using…”, “… can be considered”

### Consensus process

The Guideline Group finalised the recommendations during web-based, structured consensus conferences on 14 February 2022 and 15 March 2022 via Zoom (Zoom, Version: 5.x [Software], Zoom Video Communications, Inc., San José, California, USA. https://zoom.us). A neutral moderator facilitated the consensus conference. Voting members of the Guideline Group were delegates of all participating professional organisations, including clinicians, emergency medical services personnel and nurses, while guideline methodologists attended in a supporting role. Members with a moderate, thematically relevant conflict of interest abstained from voting on recommendations, members with a high, relevant conflict of interest were not permitted to vote or participate in the discussion. Attempts to recruit patient representatives were unsuccessful. A member of the expert group presented recommendations. Following discussion, the Guideline Group refined the wording of the recommendations and modified the grade of recommendation as needed. Agreement with both the wording and the grade of recommendation was assessed by anonymous online voting using the survey function of Zoom. Abstentions were subtracted from the denominator of the agreement rate. Consensus strength was classified as shown in Table 3.

**Table 3:** Classification of consensus strength

Description	Agreement rate
Strong consensus	> 95% of participants
Consensus	> 75 to 95% of participants
Majority approval	> 50 to 75% of participants
No approval	< 50% of participants

Recommendations were accepted if they reached consensus or strong consensus. For consensus recommendations with ≤ 95% agreement, diverging views by members of the Guideline Group were detailed in the background texts. Recommendations with majority approval were returned to the expert group for revision and further discussion at a subsequent consensus conference. Recommendations without approval were considered rejected.

### External review

During a four-week consultation phase, the recommendations and background texts were submitted to all participating professional organisations for review. Comments were collected using a structured review form. The results were then assessed, discussed and incorporated into the text by the guideline coordinator with the relevant author group.

The guideline was adopted by the executive board of the German Trauma Society on 17 January 2023.

### Quality assurance

The guideline recommendations were reviewed for consistency between guideline topic areas by the steering group. Where necessary, changes were made in collaboration with the clinical leads for all topic areas concerned. The final guideline document was checked for errors by the guideline chair and methodologist.

## Results

**Fig. 1:**
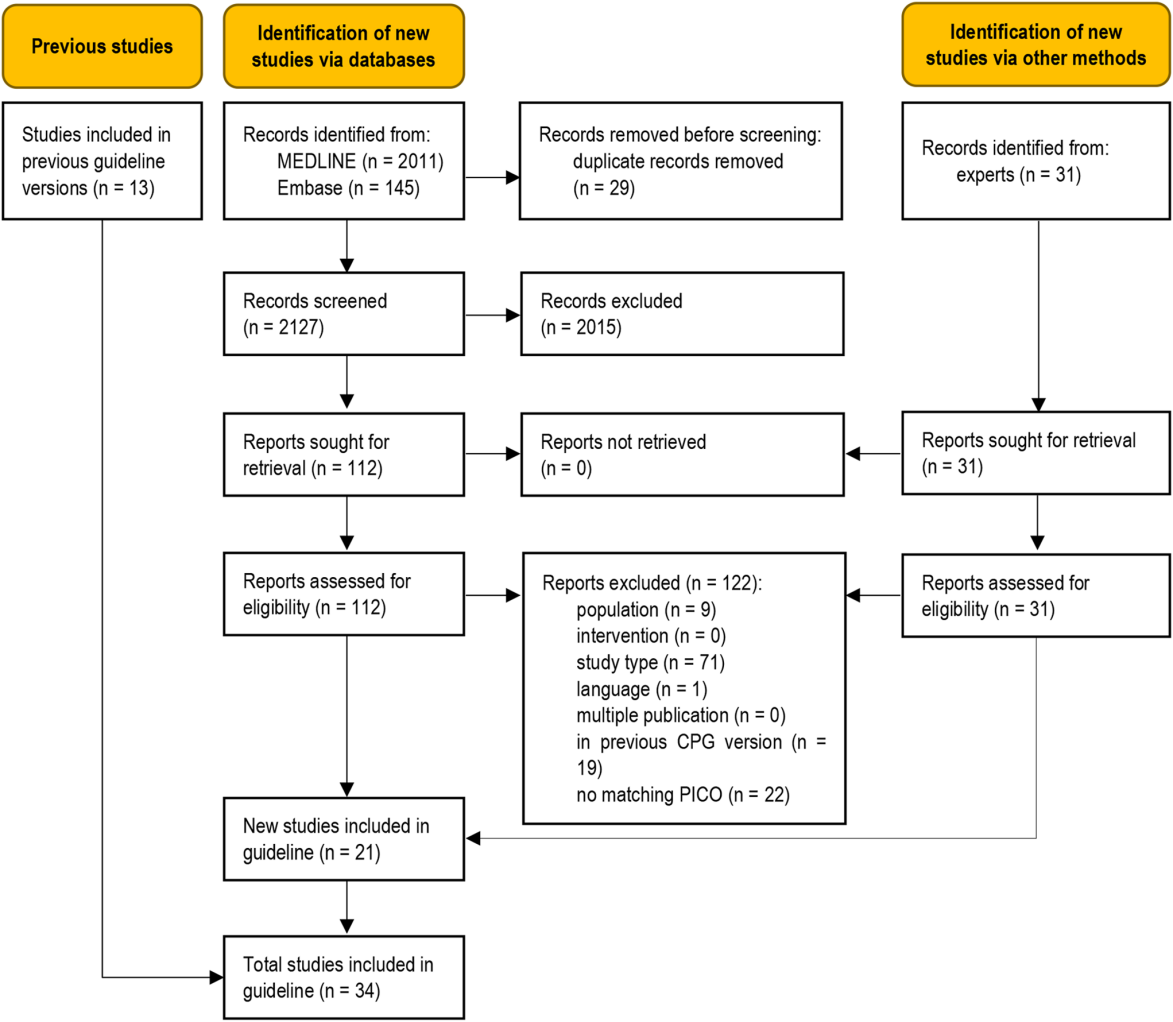
Modified PRISMA 2020 flow diagram showing the systematic literature search and selection of studies

The database searches identified 2127 unique records (Fig. 1). Additional records were obtained from clinical experts. Twenty-one new studies were eligible for this update [25–45], adding to the body of evidence from the thirteen studies previously included in the guideline [46–58]. A total of 122 full-text articles were excluded (Table S3, Online Resource 1).

### Characteristics of studies included in this update

Study characteristics, main outcomes and levels of evidence are presented in Table 4. Full details are provided in Table S4, Online Resource 1. This update included one randomised controlled trial [29], 19 prognostic cross-sectional studies [25–28, 30–42, 44, 45], and one secondary analysis of a prognostic cross-sectional study [43]. All studies, with the exception of one study on training, addressed prognostic criteria for the identification of trauma patients who are severely injured and/or require trauma team activation / trauma care. Fourteen studies were performed in North America, six in Europe, and one in India. Eligible patient populations were adults with (suspected) severe or multiple injuries or orthopaedic trainees in the study addressing staffing requirements for the resuscitation room. Some studies investigated subpopulations, e.g. trauma team activation criteria for geriatric patients [26, 36, 40, 45].

**Table 4:** Characteristics of studies included in the update (see Table S4, online resource 1 for details)

Study, ref, design, LoE, comments	Activation criterion / comparison, N, main results
Vital signs	
**Systolic blood pressure**	
Bieler 2021 [25], prognostic cross-sectional study, LoE: 2b	*SBP < 90 mmHg (N = 11, 212)* • Accurate prediction of mortality: 29.6%
Brown 2016 [26], prognostic cross-sectional study, LoE: 2b	*Step 1 or Step 2 NTTP criteria*^*a*^*using SBP < 90 mmHg vs. Step 1 or Step 2 NTTP criteria using SBP < 110 mmHg (N = 1*,*555*,*944 overall*^*b*^*)* • Undertriage^c^ reduction by substituting an SBP < 110 mmHg: 4.4% for geriatric cohort; 4.3% for adult cohort • Overtriage^c^ increase by substituting an SBP < 110 mmHg: 4.3% for geriatric cohort; 5.3% for adult cohort
Damme 2016 [28], prognostic cross-sectional study, LoE: 2b	*SBP ≤ 110 mmHg (N = 81) vs. SBP > 110 mmHg (N = 206)* • Significantly more ICU admissions, longer ICU length of stay, more ventilator days, more packed red blood cells and higher ISS for SBP ≤ 110 mmHg
Dehli 2016 [30], prognostic cross-sectional study, LoE: 3b, underpowered	*SBP < 90 mmHg (N = 9)* • Accurate prediction of ISS > 15: 56% • Accurate prediction of need for emergency procedure^c^: 44%
Guyette 2015 [31], prognostic cross-sectional study, LoE: 2b	*SBP per 5 mmHg*,* (N = 387 overall*^*b*^*)* • Lower need for resuscitative care (not significant)
Hasler 2011 [32], prognostic cross-sectional study, LoE: 2b	*Comparison of SBP intervals in blunt major trauma patients (N = 47*,*927 overall*^*b*^*)* • Significant increase in mortality in patients with SBP < 110 mmHg, linear trend
Hasler 2012 [33], prognostic registry study, LoE: 2b	*Comparison of SBP intervals in penetrating major trauma patients (N = 3444 overall* ^*b*^ *)* • Significant increase in mortality in patients with SBP < 110 mmHg, significant linear trend
Hranjec 2012 [35], prognostic cross-sectional study, LoE: 2b	*Comparison of SBP intervals (N = 57*,*973 overall*^*b*^*)* • Significantly higher mortality for SBP 0–60 mmHg and 60–90 mmHg vs. 90–120 mmHg (reference) • Significantly lower mortality for SBP 120–150 mmHg and 150–180 mmHg vs. 90–120 mmHg (reference)
Singh 2014 [42], prognostic cross-sectional study, LoE: 2b	*SBP < 90 mmHg vs. SBP > 90 mmHg (N = 9860 overall* ^*b*^ *)* • Significantly higher mortality for SBP < 90 mmHg
Tignanelli 2018 [44], prognostic cross-sectional study and comparative registry study, LoE: 2b	*SBP ≤ 90 mmHg (N = 1346)* • Accurate prediction of need for emergency procedure^c^: 63%
**Diastolic blood pressure**	
Singh 2014 [42], prognostic cross-sectional study, LoE: 2b	*DBP < 60 mmHg vs. DBP > 60 mmHg (N = 9860 overall* ^*b*^ *)* • Significantly higher mortality for DBP < 60 mmHg
**Heart rate**	
Singh 2014 [42], prognostic cross-sectional study, LoE: 2b	*HR > 120 bpm vs. HR < 120 bpm (N = 9860 overall* ^*b*^ *)* • Significantly higher mortality for HR > 120 bpm
Dehli 2016 [30], prognostic cross-sectional study, LoE: 3b, underpowered	*HR > 130 bpm (N = 3)* • Accurate prediction of ISS > 15: 0% • Accurate prediction of need for emergency procedure^c^: 67%
**Airway obstruction**,** stridor**	
Dehli 2016 [30], prognostic cross-sectional study, LoE: 3b↓, underpowered	*Airway obstruction*,* stridor (N = 4)* • Accurate prediction of ISS > 15: 50% • Accurate prediction of need for emergency procedure^c^: 75%
**Respiratory rate**	
Bieler 2021 [25], prognostic cross-sectional study, LoE: 2b	*Respiratory rate < 9 or > 29/min (N = 3207)* • Accurate prediction of mortality: 45.3%
Dehli 2016 [30], prognostic cross-sectional study, LoE: 3b↓, underpowered	*Respiratory rate > 30/min (N = 14)* • Accurate prediction of ISS > 15: 71% • Accurate prediction of need for emergency procedure^c^: 21%
**Saturation of peripheral oxygen**	
Bieler 2021 [25], prognostic cross-sectional study, LoE: 2b	*Saturation of peripheral oxygen < 90% (N = 9484)* • Accurate prediction of mortality: 31.5%
**Glasgow Coma Scale**	
Bieler 2021 [25], prognostic cross-sectional study, LoE: 2b	*GCS score < 9 (N = 15*,*099)* • Accurate prediction of mortality: 37.5% *Drop in GCS of 2 points or more (N = 3706)* • Accurate prediction of mortality: 12.9%
Dehli 2016 [30], prognostic cross-sectional study, LoE: 3b↓, underpowered	*GCS score < 13 (N = 87)* • Accurate prediction of ISS > 15: 38% • Accurate prediction of need for emergency procedure^c^: 32%
Guyette 2015 [31], prognostic cross-sectional study, LoE: 2b	*Initial GCS score per increment of 1 (N = 387 overall* ^*b*^ *)* • No detectable difference in need for resuscitative care^c^
Hranjec 2012 [35], prognostic cross-sectional study, LoE: 2b	*Comparison of different motor GCS scores (N = 57*,*973 overalll*^*b*^*)* • Significantly higher mortality for motor GCS scores of 1 and 2–5 compared to 6
Tignanelli 2018 [44], prognostic cross-sectional study and comparative registry study, LoE: 2b	*GCS score < 9 (N = 2475)* • Accurate prediction of need for emergency procedure^c^: 92%
**Body temperature**	
Bieler 2021 [25], prognostic cross-sectional study, LoE: 2b	*Hypothermia < 35 °C (N = 3040)* • Accurate prediction of mortality: 28.9%
Dehli 2016 [30], prognostic cross-sectional study, LoE: 3b↓, underpowered	*Hypothermia (core temperature < 32 °C) (N = 11)* • Accurate prediction of ISS > 15: 27% • Accurate prediction of need for emergency procedure^c^: 18%
Hranjec 2012 [35], prognostic cross-sectional study, LoE: 2b	*Comparison of temperatures (N = 57*,*973 overalll*^*b*^*)* • Significantly higher mortality for temperatures between 65-97.7 °[F] vs. 97.7-101.3 °[F] • Higher mortality for temperatures above 101.3 °[F] compared to 97.7-101.3 °[F] (not significant)
**Shock index**	
Bieler 2021 [25], prognostic cross-sectional study, LoE: 2b	*Shock index > 0.9 (N = 17*,*720)* • Accurate prediction of mortality: 17.9%
Guyette 2015 [31], prognostic cross-sectional study, LoE: 2b	*Shock index per increment of 0.1 (N = 387 overall* ^*b*^ *)* • Significantly higher need for resuscitative care^c^
Singh 2014 [42], prognostic cross-sectional study, LoE: 2b	*Shock index < 0.5 vs. >0.5* • No detectable difference for mortality *Shock index < 0.9 vs. >0.9* • No detectable difference for mortality
**Modified shock index**	
Singh 2014 [42], prognostic cross-sectional study, LoE: 2b	*Modified shock index < 0.7 vs. >0.7* • Significantly higher mortality for modified shock index < 0.7 *Modified shock index < 1.3 vs. >1.3* • Significantly higher mortality for modified shock index > 1.3
**Lactate**	
Guyette 2015 [31], prognostic cross-sectional study, LoE: 2b	*Point-of-care lactate ≥ 2.5 mmol/L (N = 228)* Need for resuscitative care^c^ associated with a 1-mmol/L difference in point-of-care lactates: • Higher within the range of < 2.5 (not significant) • Significantly higher within the range of 2.5–3.9 • Not detectable within the range of ≥ 4.0
St. John 2018 [43], secondary analysis of a prognostic cross-sectional study, LoE: 3b↓	*Prehospital lactate (N = 314 overall* ^*b*^ *)* Need for resuscitative care^c^ associated with a 1 mmol/L difference in prehospital lactate concentration: • Higher within the range of < 2.5 (not significant) • Significantly higher within the range of 2.5-4.0 • Significantly higher within the range of ≥ 4.0

Type and extent of injuries	
Dehli 2016 [30], prognostic cross-sectional study, LoE: 3b↓, underpowered	*Flail chest (N = 2)* • Accurate prediction of ISS > 15: 50% • Accurate prediction of need for emergency procedure^c^: 0% *Unstable fracture of the pelvis / fracture in two or more long bones (N = 5)* • Accurate prediction of ISS > 15: 40% • Accurate prediction of need for emergency procedure^c^: 0% *Injury in two or more body regions (head/neck/chest/abdomen/pelvis/femur/back) (N = 61)* • Accurate prediction of ISS > 15: 15% • Accurate prediction of need for emergency procedure^c^: 13% *Paralysis (N = 10)* • Accurate prediction of ISS > 15: 80% • Accurate prediction of need for emergency procedure^c^: 10% *Penetrating injury of the head/neck/chest/abdomen/pelvis/groin/back (N = 5)* • Accurate prediction of ISS > 15: 0% • Accurate prediction of need for emergency procedure^c^: 60% *Second-degree or third-degree burn injury > 15% of body surface (N = 5)* • Accurate prediction of ISS > 15: 40% • Accurate prediction of need for emergency procedure^c^: 60% *Burn injury with inhalation injury (N = 5)* • Accurate prediction of ISS > 15: 40% • Accurate prediction of need for emergency procedure^c^: 40%
Lin 2012 [39], prognostic cross-sectional study, LoE: 2b	*Two or more long bone fractures (humerus*,* radius*,* ulna*,* femur*,* tibia*,* fibula) (N = 37)* • Overtriage^c^: 29.7% • Significant prediction of ISS ≥ 25 • Significant prediction of need for an emergency operation
Tignanelli 2018 [44], prognostic cross-sectional study and comparative registry study, LoE: 2b	*Central gunshot wound (N = 1931)* • Accurate prediction of need for emergency procedure^c^: 67%

Mechanism of injury	
Dehli 2016 [30], prognostic cross-sectional study, LoE: 3b↓, underpowered	*Ejected from vehicle (N = 6)* • Accurate prediction of ISS > 15: 67% • Accurate prediction of need for emergency procedure^c^: 0% *Death of another passenger in the vehicle (N = 5)* • Accurate prediction of ISS > 15: 40% • Accurate prediction of need for emergency procedure^c^: 0% *Trapped in wreck (N = 9)* • Accurate prediction of ISS > 15: 33% • Accurate prediction of need for emergency procedure^c^: 11% *Pedestrian or cyclist hit by motor vehicle (N = 15)* • Accurate prediction of ISS > 15: 13% • Accurate prediction of need for emergency procedure^c^: 13% *Fall from > 5 m (N = 20)* • Accurate prediction of ISS > 15: 50% • Accurate prediction of need for emergency procedure^c^: 15% *Avalanche accident (N = 1)* • Accurate prediction of ISS > 15: 0% • Accurate prediction of need for emergency procedure^c^: 0%
Matsushima 2016 [40], prognostic cross-sectional study, LoE: 2b	*Motor vehicle intrusion (MVI) and age ≥ 65 years (N = 288)* • Significantly higher mortality for MVI and age ≥ 65 years *Motor vehicle intrusion (MVI) and male sex (N = 2259)* • No significantly higher mortality for MVI and male sex *Motor vehicle intrusion (MVI) and no airbag deployment (N = 300)* • No significantly lower mortality for MVI and no airbag deployment *Motor vehicle intrusion (MVI) and use of seat belt (N = 3254)* • No significantly lower mortality for MVI and use of seat belt *Motor vehicle intrusion (MVI) and heart rate > 100 bpm (N = 1175)* • Significantly higher mortality for MVI and heart rate > 100 bpm *Motor vehicle intrusion (MVI) and SBP < 110 mmHg (N = 251)* • Significantly higher mortality for MVI and SBP < 110 mmHg

Interventions	
**Airway assistance**	
Bieler 2021 [25], prognostic cross-sectional study, LoE: 2b	*Advanced airway management (N = 22*,*771)* • Accurate prediction of mortality: 27.0%
Guyette 2015 [31], prognostic cross-sectional study, LoE: 2b	*Any airway / bag valve mask attempted (N = 387overall* ^*b*^ *)* • Significantly higher need for resuscitative care^c^
Hranjec 2012 [35], prognostic cross-sectional study, LoE: 2b	*Mechanical ventilation (N = 57*,*973 overall*^*b*^*)* • Significantly higher mortality in ventilated patients
Lin 2012 [39], prognostic cross-sectional study, LoE: 2b	*Active airway assistance beyond supplemental O*_*2*_ *(N = 40)* • Overtriage^c^: 15% • Significant prediction of ISS ≥ 25 • Significant prediction of emergency operation
Tignanelli 2018 [44], prognostic cross-sectional study and comparative registry study, LoE: 2b	*Intubation (N = 3459)* • Accurate prediction of need for emergency procedure^c^: 100%
**Other interventions**	
Bieler 2021 [25], prognostic cross-sectional study, LoE: 2b	*Cardiopulmonary resuscitation (N = 3162)* • Accurate prediction of mortality: 76.2% *Insertion of a chest tube (N = 8823)* • Accurate prediction of mortality: 23.0% *Administration of catecholamines (N = 13*,*150)* • Accurate prediction of mortality: 35.7%

Combined criteria	
Lin 2012 [39], prognostic cross-sectional study, LoE: 2b	*Consciousness: BMR < 5 or paralysis or suspicion of spinal cord injury or loss of sensation or GCS score ≤12 (N = 128)* • Overtriage^c^: 29.7% • Significant prediction of ISS ≥ 25 • Significant prediction of need for an emergency operation *Circulation: no radial pulse and sustained heart rate ≥ 120 bpm*,* or SBP ≤ 90 mmHg (N = 63)* • Overtriage^c^: 31.7% • Significant prediction of ISS ≥ 25 • Significant prediction of need for an emergency operation *Cutaneous: deep penetrating injury to head*,* neck & torso*,* amputation at or proximal to wrist or ankle (N = 139)* • Overtriage^c^: 52.5% • No significant prediction of ISS ≥ 25 • Significant prediction of need for an emergency operation^c^ *Two or more level two criteria met (age > 55 years*,* respiratory rate ≥ 30/min*,* BMR = 5*,* sustained heart rate = 120 bpm*,* long bone fracture sustained in a motor vehicle collision or fall ≥ 10 feet*,* major degloving injury*,* or major flap avulsion > 5 inches*,* or gunshot wound to the extremities) (N = 44)* • Overtriage^c^: 72.7% • No significant prediction of ISS ≥ 25
Dehli 2016 [30], prognostic cross-sectional study, LoE: 3b↓, underpowered	*Traumatic amputation or crush injury above wrist/ankle (N = 1)* • Accurate prediction of ISS > 15: 0% • Accurate prediction of need for emergency procedure^c^: 100%
Cull 2019 [27], prognostic cross-sectional study, LoE: 2b	*Trauma activation prediction models (mathematical equations including SBP*,* PR*,* RR*,* GCS) for falls*,* gunshot wounds and stab wounds*,* (N = 157*,*164 overall*^*b*^*)* • Accurate prediction of trauma activation level: approximately 52% (gunshot wounds) 59% (falls) 75% (stab wounds)
Guyette 2015 [31], prognostic cross-sectional study, LoE: 2b	*Point-of-care lactate ≥ 2.5 mmol/L and SBP 91–100 mmHg (N = 93)* • Accurate prediction of need for resuscitative care^c^: 22.6%
Heindl 2021 [34], prognostic cross-sectional study, LoE: 2b	*GoR A criteria (N = 32) vs. GoR B criteria (N = 84) (according to the German Polytrauma Guideline of 2016) vs. GoR 0 criteria (N = 48) (TTA based on the emergency physician’s assessment alone)* • Significantly higher mortality for GoR A criteria • More emergency interventions for GoR A criteria
Kalkwarf 2021 [37], prognostic cross-sectional study, LoE: 3b↓, post-hoc analysis	*≥ 2 ABC criteria (N = 25) (penetrating trauma*,* heart rate > 120 bpm*,* SBP < 90 mmHg*,* positive abdominal FAST) vs. <2 ABC criteria (N = 266)* • Significantly higher mortality with ≥ 2 criteria met
Lehmann 2009 [38], prognostic cross-sectional study, LoE: 2b	*Institution’s current triage system based on three steps (vital signs and level of consciousness*,* anatomy of injury*,* biomechanics of injury and other risk factors) vs. simplified triage protocol using four variables (SBP < 100 mmHg*,* GCS < 14*,* altered respirations*,* penetrating truncal injury) (N = 244 overall*^*b*^*)* • Significantly better prediction of need for emergency intervention with simplified criteria; significant differences between different steps of current criteria • Negative predictive value of need for emergency intervention: 99.6% with current system, 96% with simplified protocol • Positive predictive value of 21% with current system and 58% with simplified protocol
Shawhan 2015 [41], prognostic cross-sectional study, LoE: 2b	*Level 1* ^*d*^ *(N = 89) vs. level 2* ^*e*^ *(N = 146) activation criteria vs. level 3 (trauma consultation) (N = 225)* • Better prediction of patients requiring ICU admission for level 1 compared to level 2 • Better prediction of patients requiring urgent intervention for level 1 compared to levels 2 or 3

Adult vs. geriatric patients / comparison of age groups	
Brown 2016 [26], prognostic cross-sectional study, LoE: 2b	*Physiologic Step 1 or anatomic Step 2 NTTP criteria using SBP < 90 mmHg vs. physiologic Step 1 or anatomic Step 2 NTTP criteria using SBP < 110 mmHg*,* (N = 1*,*555*,*944 overalll*^*b*^*)* • Undertriage^c^ reduction by substituting an SBP < 110 mmHg: 4.4% for geriatric cohort; 4.3% for adult cohort • Overtriage^c^ increase by substituting an SBP < 110 mmHg: 4.3% for geriatric cohort; 5.3% for adult cohort
Hranjec 2012 [35], prognostic cross-sectional study, LoE: 2b	*Comparison of different age groups (N = 57*,*973 overall*^*b*^*)* • Significantly higher mortality for > 85 years vs. 65 years (reference) • Significantly higher mortality for 65–75 years vs. 65 years (reference)
Ichwan 2014 [36], prognostic cross-sectional study, LoE: 2b	*Standard adult triage criteria*^*f*^*vs. geriatric triage criteria*^*g*^*(age ≥ 70) (N = 101*,*577 overall*^*b*^*)*: • Geriatric triage criteria more sensitive for prediction of ISS > 15 compared to standard adult triage criteria for both, adults and geriatrics • Geriatric triage criteria less specific for prediction of ISS > 15 compared to standard adult triage criteria for both, adults and geriatrics
Wermann 2011 [45], prognostic cross-sectional study, LoE: 3b↓, indirectness	*GCS = 14 (geriatric patients > 70 years) vs. GCS = 13 (adult patients) (N = 90*,*597 overall*^*b*^*)*: • Significantly higher mortality in geriatric patients *SBP 91–100 mmHg (geriatric patients > 70 years) vs. SBP 81–90 mmHg (adult patients)*: • Trend towards comparable mortality *Significantly higher mortality in geriatric patients (> 70 years) vs. adult patients on the basis of*: • fall with traumatic brain injury, pedestrian struck by vehicle, multiple body system injuries, fracture of humerus or femur from motor vehicle *Non-significant trend towards higher mortality in geriatric patients (> 70 years) vs. adult patients on the basis of*: • fall with traumatic chest injury or traumatic spinal cord injury
Matsushima 2016 [40], prognostic cross-sectional study, LoE: 2b	*Motor vehicle intrusion in patients < 18 years vs. patients between 19 and 64 years vs. patients ≥ 65 years*,* (N = 3998 overall*^*b*^*)* • Significantly higher mortality in patients ≥ 65 years of age

Staffing– training	
Daurka 2015 [29], RCT, LoE: 2b↓, unclear risk of bias, underpowered	*Pelvic training + an introduction to the ABC algorithm (N = 11) vs. pelvic training alone (N = 9)* • The ABC teaching concept yielded improvements in coagulopathy assessment and management, urological injury, bowel injury / open fracture assessment, and appropriate prioritisation

^**a**^ Step 1 NTTP criteria (GCS score ≤ 13, SBP < 90 mm Hg, respiratory rate [RR] < 10 or RR > 29), Step 2 criteria (penetrating injury, flail chest, open skull fracture, ≥ 2 proximal long bone fractures, pelvic fracture, crush injury, amputation, paralysis); ^b^overall: Number of cases only available for the entire study population and not for individual activation criteria ^**c**^ for definitions of undertriage, overtriage and emergency procedures see Table S4, Online Resource 1; ^**d**^ level 1: hypotension (SBP ≤ 90 mmHg), GCS < 13 (currently), penetrating injury to neck, chest, or abdomen, altered respirations or intubation in the field, proximal extremity amputation, multiple incoming patients with severe injuries; ^**e**^ level 2: GCS 13–14, pulse > 12, mangled extremity or distal amputation, age > 65 years + mechanism, neurologic deficit, burns > 20% BSA or inhalation, multiple long bone fractures or mangled extremity, flail chest, peritonitis on abdominal exam, pregnancy; ^**f**^ SBP < 90 mmHg, or radial pulse absent with carotid pulse present, GCS ≤ 13, fractures of ≥ 2 proximal long bones; ^**g**^ SBP < 100 mmHg or radial pulse absent with carotid pulse present, GCS ≤ 14 in trauma patient with a known or suspected traumatic brain injury, fracture of one proximal long bone sustained in a motor vehicle crash, injury to ≥ 2 body regions, pedestrian struck by motor vehicle, fall from any height including standing falls with evidence of traumatic brain injury

For abbreviations and acronyms see list included

### Risk-of-bias assessment for included studies and levels of evidence

The risk of bias for prognostic cross-sectional studies was not systematically assessed since no relevant risk-of-bias tool had been predefined in the guideline methods. The risk of selection bias was judged to be high for a comparison of interventions. The risk of bias was unclear in one randomised controlled trial of different training options for orthopaedic trainees.

The LoE was downgraded for five studies. Reasons for downgrading were post-hoc secondary analyses (two studies), low power (two studies), and indirectness (one study).

### Recommendations

One recommendation was confirmed, two recommendations were modified, and six new recommendations were developed based on the updated evidence and expert consensus (Table 5). Three recommendations from the 2016 Guideline were not retained in the 2022 update (Table S5, Online Resource 1).

**Table 5:** List of recommendations with grade of recommendation and strength of consensus

No.	GoR	New evidence, consensus^a^	Recommendation	Status 2022	
*Trauma team staffing requirements*					
1	A ⇑⇑	– 100%	Use fixed teams (trauma teams) to provide care to severely injured patients on the basis of pre-structured plans and/or ensure they have completed special training	Confirmed	
2	GPP	– 100%	Ensure that an inter-professional trauma team consists of at least two nurses and at least two physicians with appropriate levels of competence in emergency medical care and emergency surgery	Modified	
3	GPP	100%	Ensure that it is possible at all times to add other specialists to the trauma team (extended trauma team) depending on the level of care provided by the hospital	Modified	
*Criteria for trauma team activation*					
4	A ⇑⇑	[25, 26, 30, 32, 33, 37, 38, 41, 42, 44, 45] 94.4%	Activate the trauma team for patients with any of the following pathological findings after trauma: Airway/breathing (A/B) problem • Respiratory problems (SpO2 < 90%) / requirement for airway management • Respiratory rate < 10 or > 29 breaths per minute Circulation (C) problem • Systolic blood pressure < 90 mmHg • Heart rate > 120 bpm • Shock index > 0.9 • Positive eFAST Disability (D) problem • GCS score ≤ 12 Exposure (E) problem • Hypothermia < 35.0 °C	New	
5	A ⇑⇑	[30, 34, 37, 38, 41, 44] 100%	Activate the trauma team for patients who present with any of the following injuries or have undergone any of the following procedures after trauma: • Flail chest • Mechanically unstable pelvic fracture • Penetrating injuries to the torso or neck region • Traumatic amputation proximal to the wrist or ankle • Sensorimotor deficit after spinal cord injury • Prehospital intervention (requirement for airway management, chest decompression, administration of catecholamines, pericardiocentesis, application of tourniquet)	New	
6	B ⇑	[30, 36] 100%	The trauma team should be activated for patients with any of the following injuries after trauma: • More than two proximal long bone fractures • Burns > 20% and ≥ 2b degree	New	
7	B ⇑	[30] 100%	The trauma team should be activated based on the following additional criteria: • Fall from a height of more than three metres • Road traffic accident with ejection from vehicle or long bone fracture	New	
8	B ⇑	[35, 45] 81.3%	Trauma teams should be activated more readily for geriatric patients	New	
9	B ⇑	[35, 36, 45] 100%	The trauma team should also be activated for geriatric patients after relevant trauma when any of the following additional criteria is met: • SBP < 100 mmHg • GCS score ≤ 14 in the presence of known or suspected traumatic brain injury • Two or more injured body regions • Any long bone fracture after road traffic accident	New	

GoR, grade of recommendation

## Discussion

### Rationale for recommendations

The problem of overtriage has already been addressed in the Introduction section. Especially criteria with moderate risk for severe injury (e.g. injury mechanism and setting) have been found to be poorly specific for the identification of severely injured patients [59].

In a retrospective study involving different resuscitation-room patient groups, Heindl et al. (2021) found that emergency interventions were necessary in only 0.6% of the cases in which a trauma team had been activated on the basis of criteria with moderate risk for severe injury. By contrast, emergency interventions were required in 75% of the cases in which trauma team activation had been based on criteria for high risk for severe injury [60].

Shawhan et al. (2015) were able to reduce the rate of overtriage from 79 to 44% by using a simplified triage system that, for example, eliminated mechanism of injury criteria [41]. Similar results were reported by Uleberg et al. (2015), who conducted a study on 809 trauma patients and found that injury mechanism and setting criteria caused an overtriage rate of 78% [61]. Matsushima et al. (2016) investigated the use of motor vehicle intrusion as the sole indicator of injury severity and the sole criterion for trauma team activation. The rate of overtriage was 85.5% in a group of 3998 patients [40]. These results were confirmed by Dehli et al. (2011) and Lavoie et al. (2010), who too reported that inclusion of the mechanism of injury criterion accounted for a large proportion of overtriage [30, 62].

In the review presented here, these research findings were incorporated into new recommendations for trauma team activation which were evaluated and approved on the basis of data from a systematic search of the literature and a subsequent inter-professional consensus process [30, 36, 63].

Criteria with high risk for severe injury were thoroughly re-evaluated, revised and modified on the basis of the systematic literature review.

Studies have shown, for example, that trauma team activation on the basis of prehospital clinical parameters is effective since these parameters are often found to be directly associated with mortality [35, 37, 38, 44, 46]. Bleeding as well as a heart rate > 120 beats per minute and a systolic blood pressure < 90 mm Hg as surrogates play an important role in this context. Kalkwarf et al. found a significant association between these parameters and procedures such as emergency laparotomy, (massive) transfusion, and relevant bleeding [44].

Trauma team activation is also recommended in the case of patients presenting with specific injury patterns or requiring specific prehospital interventions [40, 42, 43, 50]. Shawhan et al. investigated a cohort of 460 patients and reported positive predictive values of up to 63% depending on the detected injury or the pathological clinical parameter. Their approach allowed overtriage to be reduced. In a high percentage of cases, clinical parameters such as flail chest, mechanically unstable pelvic fracture, and sensorimotor deficit after spinal cord injury are associated with an Injury Severity Score < 15 and, together with other modifications, have therefore been included as new criteria with high risk for severe injury in the revised list of trauma activation criteria.

Dehli et al. reported that a fall from a height > 5 m and ejection from a vehicle predicted severe injuries (ISS > 15) with a positive predictive value of at least 50%. For this reason, ejection from vehicle and fall from height continue to be criteria with moderate risk for severe injury. By contrast, frontal collision and intrusion of more than 50–75 cm, collision involving a pedestrian or cyclist, and death of a passenger in a vehicle were removed from the list of criteria. Several authors [52–54] reported high rates of overtriage when mechanistic criteria alone were used to describe injury severity.

The recommendations for geriatric patients can be regarded as additional criteria for trauma team activation. These patients have so far not been adequately studied and addressed in the management of severely injured patients. As a result of higher levels of physical activity in the elderly and also as a result of an aging population, the number of serious accidents involving elderly people is likely to increase. Since the bodily compensation and response mechanisms in elderly patients are different from those in younger patients, trauma teams should be activated more readily for geriatric patients [35, 45, 63].

Studies from the United States reported that geriatric patients had a three to five times higher rate of mortality than non-geriatric patients despite similar injury severity [35, 45]. The authors of these studies came to the conclusion that these elderly patients should be transported to a level I trauma centre for diagnosis and treatment. Werman et al. (2011) used trauma registry data on more than 90,000 patients and developed geriatric-specific criteria for trauma team activation [45]. They identified a number of criteria that demonstrated a significant increase in the mortality risk for patients ≥ 70 years compared with patients < 70 years. Werman et al. concluded that geriatric patients should be transported to a trauma centre if they met any of the following criteria: (a) GCS score ≤ 14, (b) systolic blood pressure < 100 mmHg, (c) specific mechanisms of injury, (d) comorbidities, and (e) the presence of a long bone fracture.

Since there are almost thirty possible relevant comorbidities, this criterion was not included in the present guideline after consultation with the Geriatric Traumatology Working Group. What is more, comorbid conditions are often difficult to evaluate at the scene of injury and the prevalence of comorbidities is likely to be disproportionately high among elderly patients and can lead to the problem of overtriage (see above). The criterion “comorbidities” will be re-evaluated in the next guideline revision.

Ichwan et al. (2014) too investigated the application of geriatric-specific triage criteria and showed that the sensitivity of non-geriatric triage criteria for severely injured patients (ISS > 15) was considerably lower for patients aged 70 years or older (61%; 95% CI 60–62%) than for patients who were older than 16 years and younger than 68 years (87%; 95% CI 86–87%) [36]. The use of geriatric criteria increased sensitivity in patients ≥ 70 years (93%; 95% CI 92–93%). For this reason, the following trauma team activation criteria for geriatric patients were included for the first time in the current guideline (see Table 5):

- Systolic blood pressure < 100 mmHg.
- GCS score ≤ 14 in the presence of known or suspected traumatic brain injury.
- Two or more injured body regions.
- Any long bone fracture after road traffic accident.

The trauma team activation criteria defined in the 2016 S3 Guideline have been revised to reflect new research and have been included in the updated guideline. Especially criteria with moderate risk for severe injury (e.g. injury mechanism and setting) have been modified. In addition, geriatric-specific trauma team activation criteria have been incorporated into this guideline for the first time. Although this is a success in principle, it must be noted that activation for, for example, a GCS < 14 in geriatric patients seems to be a very common criterion, even if it is in line with the current literature. As staff and resources in the healthcare system are increasingly a problem, this can lead to rationing and difficult triage assessments. Therefore, continuous work on improving criteria for TTA must be continued in the future. Perhaps in the future, the concept of TTA based on individual parameters (e.g. blood pressure) will be abandoned and criteria patterns instead of single criteria will be implemented. This could be extremely helpful for geriatric patients with relevant comorbidities, especially if supported by AI.

### Limitations of the guideline

Patient values and preferences were sought but not received. The effect of this on the guideline is unclear, and there is a lack of research evidence on the effect of patient participation on treatment decisions or outcomes in the emergency setting.

### Unanswered questions and future research

The trauma team activation criteria presented here only reflect new research and the most current literature as well as the expert group’s clinical experience. The effectiveness of the changes and updates made to the guideline will now be tested in clinical practice and in the everyday management of severely injured patients. An essential prerequisite is the trauma registry of the German Trauma Society, which contains extensive data that helps to answer many important and interesting questions. It will be interesting to assess the effectiveness of the new trauma team activation criteria for geriatric patients, which have not yet been included in resuscitation room algorithms. Further research must be conducted to determine whether and, if so, to what extent the use of these criteria leads to overtriage. The new criteria with moderate risk for severe injury will have to be re-evaluated as well.

## Electronic supplementary material

Below is the link to the electronic supplementary material.

Supplementary Material 1

## Data Availability

No datasets were generated or analysed during the current study.

## Abbreviations

AWMF: German Association of the Scientific Medical Societies
BMR: Best motor response
Bpm: Beats per minute
BSA: Body surface area
CI: Confidence interval
CT: Computed tomography
DBP: Diastolic blood pressure
ED: Emergency department
GCS: Glasgow Coma Scale
GoR: Grade of recommendation
GPP: Good (clinical) practice point
HR: Heart rate
ICU: Intensive care unit
ISS: Injury Severity Score
LoE: Level of evidence
MVI: Motor vehicle intrusion
N: Number
NTTP: National Trauma Triage Protocol
PICO: Population, intervention, comparison, outcome
PRISMA: Preferred Reporting Items for Systematic Reviews and Meta-Analyses
RCT: Randomised controlled trial
RR: Respiratory rate
SBP: Systolic blood pressure
Y: Years
