# Supplementary material for: Criteria for trauma team activation and staffing requirements for the management of patients with (suspected) multiple and/or severe injuries in the resuscitation room– a systematic review and clinical practice guideline update
Source: Eur J Trauma Emerg Surg. 2025 Mar 18;51(1):142. doi: 10.1007/s00068-025-02817-7 (PMC11920003; doi:10.1007/s00068-025-02817-7)
Supplement: Supplementary file 1 — Supplementary Material 1 [file 68_2025_2817_MOESM1_ESM.docx]

2.3 Trauma team activation criteria and staffing components in the emergency room

# S1 PICO Questions

|  | Population | Intervention(s) | Control(s) | Outcome(s) |
| --- | --- | --- | --- | --- |
| 1 | Potentially severely injured or polytraumatised patients | Trauma team activation for specific criteria/exposures. | Comparison to no trauma team activation different levels of emergency care  or  trauma team activation for other criteria/exposures | Patient-relevant outcomes |
| 2 | Potentially severely injured or polytraumatised patients | Comparison of different criteria/exposures for predicting severely injured patients or the need for emergency intervention (in the context of trauma team activation/trauma triage) | Comparison of different criteria/exposures for predicting serious injuries or the need for emergency intervention (in the context of trauma team activation/trauma triage). | Patient-relevant outcomes or quality criteria for the prediction of severely injured patients or the need for emergency interventions (e.g. sensitivity, specificity). |
| 3 | Potentially severely injured or polytraumatised patients | Care provided by fixed teams that work according to pre-structured plans and/or have undergone special training | open | Patient-relevant outcomes |
| 4 | Practitioners (doctors, nurses) working in the care of polytrauma or severely injured patients | Training for emergency care  or  Pre-structured plans for trauma teamcare | open | (Patient-relevant) test results |
| 5 | Potentially severely injured or polytrauma patients | Care by a trauma team with a defined composition | open | Patient-relevant outcomes |
| 6 | Potentially severely injured or polytrauma patients | Extended trauma teams are kept on hand depending on the level of care provided | open | Patient-relevant outcomes |
| 7 | Potentially severely injured or polytrauma patients | Additional care by physicians who are present within a defined time window according to their request | open | Patient-relevant outcomes |
| 8^§^ | Potentially severely injured or polytrauma patients | Registration of shock room B without emergency physician | Shock room-B registration with emergency physician | Treatment capacity overload in the emergency department/patient-relevant outcomes |

^§^ new question

# S2 Literature Search

| Suchstrategie 2021, MEDLINE (via Ovid) Datum: 27.08.2021 2.011 Treffer |
| --- |
| 1. (trauma team adj2 (activation? or requirement)).ab,ti,kf.  2. (trauma room or shock room or resuscitation area? or resuscitation room or emergency room or reanimation room or trauma bay).ti,ab,kf. and (exp Triage/ or (triage or patient selection).ab,ti,kf.)  3. exp Trauma Centers/og  4. ((trauma room or shock room or resuscitation area? or resuscitation room or emergency room or reanimation room or trauma bay) and (team? or surgeon? or physician?)).ab,ti,kf.  5. (trauma team or trauma surgeon? or trauma physician?).ab,ti,kf. and (response time* or ((in-house or on-call) adj1 attend*)).ti,ab,kf.  6. 1 or 2 or 3 or 4 or 5  7. exp Multiple Trauma/  8. (polytrauma* or trauma patient? or (severe adj2 shock)).ti,ab,kf.  9. ((multiple or major or severe* or serious*) adj3 (trauma* or injur*)).ti,ab,kf.  10. ((blunt or penetrating) adj5 (trauma* or injur*)).ti,ab,kf.  11. (*Critical Care/ or *Emergencies/ or (life threatening or critical* care or emergen*).ti,ab,kf.) and (trauma* or injur*).ti,ab,kf.  12. 7 or 8 or 9 or 10 or 11  13. 6 and 12  14. exp animals/ not humans.sh.  15. 13 not 14  16. (comment or editorial or letter).pt. or case report*.mp.  17. 15 not 16 |
| Suchstrategie 2021, Embase (via Elsevier) Datum: 27.08.2021 145 Treffer |
| #1 (“trauma team” NEAR/2 (activation? OR requirement)):ti,ab,kw  #2 ("trauma room" OR "shock room" OR "resuscitation area?" OR "resuscitation room" OR "emergency room" OR “reanimation room” OR “trauma bay”):ti,ab,kw AND ('emergency health service'/exp OR (triage OR “patient selection”):ti,ab,kw)  #3 ((“trauma room” OR “shock room” OR “resuscitation area?” OR “resuscitation room” OR “emergency room” OR “reanimation room” OR “trauma bay”) AND (team? or surgeon? or physician?)):ti,ab,kw  #4 (“trauma team” OR “trauma surgeon?” OR “trauma physician?”):ti,ab,kw AND (“response time*” OR ((“in-house” OR “on-call”) NEXT/1 attend*)):ti,ab,kw  #5 #1 OR #2 OR #3 OR #4  #6 'multiple trauma'/exp  #7 (polytrauma* OR "trauma patient?"):ti,ab,kw OR (severe NEXT/2 shock):ti,ab,kw  #8 ((multiple OR major OR severe* OR serious*) NEXT/3 (trauma* OR injur*)):ti,ab,kw  #9 ((blunt OR penetrating) NEXT/5 (trauma* OR injur*)):ti,ab,kw  #10 ('intensive care'/mj OR 'emergency'/mj OR ("life threatening" OR "critical care" OR emergen*):ti,ab,kw) AND (trauma* OR injur*):ti,ab,kw  #11 #6 OR #7 OR #8 OR #9 OR #10  #12 #5 AND #11  #13 'animals'/exp NOT 'humans'/de  #14 #12 NOT #13  #15 (comment OR editorial OR letter):it OR "case report*":ti,ab,kw  #16 #14 NOT #15  #17 [embase]/lim  #18 #16 AND #17  #19 embase NOT (embase AND medline)  #20 #18 AND #19  #21 #20 AND ('article'/it OR 'article in press'/it OR 'review'/it) |

# S3 Excluded Studies

| **Reference** | **Reason for exclusion** |
| --- | --- |
| Ahmed, J. M., et al. (2007). "Trauma management outcomes associated with nonsurgeon versus surgeon trauma team leaders." Annals of Emergency Medicine 50(1): 7-12, 12.Study population1. | Study type |
| Amiel, I., et al. (2016). "Mobile in Situ Simulation as a Tool for Evaluation and Improvement of Trauma Treatment in the Emergency Department." Journal of Surgical Education 73(1): 121-128. | Study type |
| Andres, R., et al. (2017). "Design and Implementation of a Trauma Care Bundle at a Community Hospital." BMJ Quality Improvement Reports 6(1). | Study type |
| Arbabi, S., et al. (2003). "Patient outcomes in academic medical centers: influence of fellowship programs and in-house on-call attending surgeon." Archives of Surgery 138(1): 47-51 | Reference was not yet considered in previous guideline |
| Armenia, S., et al. (2018). "The Role of High-Fidelity Team-Based Simulation in Acute Care Settings: A Systematic Review." The Surgery Journal 4(3): Study population36-Study population51. | Study type |
| Bang, M., et al. (2018). "Validation of the Korean criteria for trauma team activation." Clinical & Experimental Emergency Medicine 5(4): 256-263. | Study type |
| Bardes, J. M., et al. (2019). "Old Age With a Traumatic Mechanism of Injury Should Be a Trauma Team Activation Criterion." Journal of Emergency Medicine 57(2): 151-155. | Study type |
| Barleycorn, D. and G. A. Lee (2018). "How effective is trauma simulation as an educational process for healthcare providers within the trauma networks? A systematic review." International emergency nursing 40: 37-45. | Study type |
| Bauman, Z. M., et al. (2020). "Rural Trauma Team Development Course Instills Confidence in Critical Access Hospitals." World Journal of Surgery 44(5): 1478-1484. | Study type |
| Baxt, W. G., et al. (1990). "The trauma triage rule: a new, resource-based approach to the prehospital identification of major trauma victims." Annals of Emergency Medicine 19(12): 1401-1406. | Reference was not yet considered in previous guideline |
| Benjamin, E., et al. (2015). "Witnessed aspiration in trauma: Frequent occurrence, rare morbidity--A prospective analysis." The Journal of Trauma and Acute Care Surgery 79(6): 1030-1036; discussion 1036-1037. | Study can be assigned to a specific recommendation or predefined question (according to PICO scheme) |
| Benjamin, E. R., et al. (2018). "The Age of Undertriage: Current Trauma Triage Criteria Underestimate The Role of Age and Comorbidities in Early Mortality." Journal of Emergency Medicine 55(2): 278-287. | Study can be assigned to a specific recommendation or predefined question (according to PICO scheme) |
| Bieler, D., et al. (2018). "[Optimization of criteria for activation of trauma teams : Avoidance of overtriage and undertriage]." Unfallchirurg 121(10): 788-793. | Study type |
| Boyle, M. J. (2007). "Is mechanism of injury alone in the prehospital setting a predictor of major trauma - a review of the literature." Journal of Trauma Management & Outcomes [Electronic Resource] 1(1): 4. | Study type |
| Bozorgi, F., et al. (2019). "Investigation of Frequency of the Lethal Triad and Its 24 Hours Prognostic Value among Patients with Multiple Traumas." Open Access Macedonian Journal of Medical Sciences 7(6): 962-966. | Study can be assigned to a specific recommendation or predefined question (according to PICO scheme) |
| Braken, P., et al. (2018). "Simple modification of trauma mechanism alarm criteria published for the TraumaNetwork DGU<sup> R</sup> may significantly improve overtriage - a cross sectional study." Scandinavian Journal of Trauma, Resuscitation & Emergency | Study type |
| Brown, J. B., et al. (2016). "Prehospital lactate improves accuracy of prehospital criteria for designating trauma activation level." The Journal of Trauma and Acute Care Surgery 81(3): 445-452. | Study type |
| Butcher, N. and Z. J. Balogh (2012). "AIS>2 in at least two body regions: a potential new anatomical definition of polytrauma." Injury 43(2): 196-199. | Study can be assigned to a specific recommendation or predefined question (according to PICO scheme) |
| Butler, M. B., et al. (2018). "Effect of an Emergency Medicine Resident as Team Leader on Outcomes of Trauma Team Activations." Aem Education & Training 2(2): 107-114. | Study can be assigned to a specific recommendation or predefined question (according to PICO scheme) |
| Butcher, N. E. and Z. J. Balogh (2013). "The practicality of including the systemic inflammatory response syndrome in the definition of polytrauma: experience of a level one trauma centre." Injury 44(1): 12-17. | Study type |
| Byars, D. V., et al. (2020). "In Situ Simulation for Ventilator Management in Emergency Medicine." Aem Education & Training 4(4): 415-418. | Study type |
| Capella, J., et al. (2010). "Teamwork training improves the clinical care of trauma patients." Journal of Surgical Education 67(6): 439-443. | Study type |
| Cherry, R. A., et al. (2010). "Outcome assessment of blunt trauma patients who are undertriaged." Surgery 148(2): 239-245. | Study type |
| Cohen, R., et al. (2012). "Involvement of surgical residents in the management of trauma patients in the emergency room: does the presence of an attending physician affect outcomes?" World Journal of Surgery 36(3): 539-547. | Study population |
| Coniglio, R., et al. (2018). "A Multicenter Performance Improvement Program Uses Rural Trauma Filters for Benchmarking: An Evaluation of the Findings." Journal of Trauma Nursing 25(2): 139-145. | Study can be assigned to a specific recommendation or predefined question (according to PICO scheme) |
| Cortez, R. (2018). "Geriatric Trauma Protocol." Journal of Trauma Nursing 25(4): 218-227. | Study population |
| Day, D. L., et al. (2020). "Correlation of Nasal Cannula End-Tidal Carbon Dioxide Concentration With Need for Critical Resources for Blunt Trauma Patients Triaged to Lower-Tier Trauma Activation." Journal of Trauma Nursing 27(2): 88-95. | Study can be assigned to a specific recommendation or predefined question (according to PICO scheme) |
| de la Mar, A. C. J., et al. (2021). "In-house versus on-call trauma surgeon coverage: A systematic review and meta-analysis." The Journal of Trauma and Acute Care Surgery 91(2): 435-444. | Study type |
| Demetriades, D., et al. (2001). "Old age as a criterion for trauma team activation." Journal of Trauma-Injury Infection & Critical Care 51(4): 754-756; discussion 756-757. | Reference was not yet considered in previous guideline |
| Dinh, M. M., et al. (2014). "Refining the trauma triage algorithm at an Australian major trauma centre: derivation and internal validation of a triage risk score." European Journal of Trauma & Emergency Surgery 40(1): 67-74. | Study type |
| Dinh, M. M., et al. (2012). "Performance of the New South Wales Ambulance Service major trauma transport protocol (T1) at an inner city trauma centre." Emergency Medicine Australasia 24(4): 401-407. | Study type |
| Engum, S. A., et al. (2000). "Prehospital triage in the injured pediatric patient." Journal of Pediatric Surgery 35(1): 82-87. | Reference was not yet considered in previous guideline |
| Eraybar, S., et al. (2019). "Comparison of Fatal Injuries Resulting from Tractor and High Speed Motorcycle Accidents in Turkey: A Multicenter Study." Emergency Medicine International Print 2019: 9471407. | Study can be assigned to a specific recommendation or predefined question (according to PICO scheme) |
| Frink, M., et al. (2010). "[Injury severity and pattern at the scene. What is the influence of the mechanism of injury?]." Unfallchirurg 113(5): 360-365. | Study population |
| Greene, W., et al. (2007). "Pregnancy is not a sufficient indicator for trauma team activation." Journal of Trauma-Injury Infection & Critical Care 63(3): 550-554; discussion 554-555. | Study type |
| Grimme, K., et al. (2005). "Calculation of different triage scores based on the German Trauma Registry: Value of the shock index." European Journal of Trauma 31(5): 480-487. | Reference was not yet considered in previous guideline |
| Hajib, et al. (2017). "Who should lead a trauma team: Surgeon or non-surgeon? A systematic review and meta-analysis." Journal of Injury & Violence Research 9(2): 107-116. | Study type |
| Hamed, R., et al. (2018). "Prognostic value of scoring tools in severe trauma patients admitted to the emergency department." Tunisie Medicale 96(3): 203-208. | Language |
| Harbrecht, B. G., et al. (2016). "Intubated Trauma Patients Do Not Require Full Trauma Team Activation when Effectively Triaged." Journal of the American College of Surgeons 222(4): 603-611. | Study type |
| Harmsen, A. M. K., et al. (2017). "Optimization of trauma care: A two-tiered inhospital trauma team response system." Archives of Trauma Research 6(3): 15-19. | Study population |
| He, K., et al. (2019). "Crash Telemetry-Based Injury Severity Prediction is Equivalent to or Out-Performs Field Protocols in Triage of Planar Vehicle Collisions." Prehospital & Disaster Medicine 34(4): 356-362. | Study can be assigned to a specific recommendation or predefined question (according to PICO scheme) |
| Helling, T. S., et al. (2003). "The presence of in-house attending trauma surgeons does not improve management or outcome of critically injured patients." Journal of Trauma-Injury Infection & Critical Care 55(1): 20-25. | Study type |
| Hong, Y. and X. Cai (2018). "Effect of team training on efficiency of trauma care in a Chinese hospital." Journal of International Medical Research 46(1): 357-367. | Study type |
| Hoyle, A. C., et al. (2020). "Undertriage of the elderly major trauma patient continues in major trauma centre care: a retrospective cohort review." Emergency Medicine Journal 37(8): 508-514. | Study can be assigned to a specific recommendation or predefined question (according to PICO scheme) |
| Hsiao, K. H., et al. (2013). "Whole-body computed tomography in the initial assessment of trauma patients: is there optimal criteria for patient selection?" Emergency Medicine Australasia 25(2): 182-191. | Study can be assigned to a specific recommendation or predefined question (according to PICO scheme) |
| Kann, S. H., et al. (2007). "Evaluation of pre-hospital trauma triage criteria: a prospective study at a Danish level I trauma centre." Acta Anaesthesiologica Scandinavica 51(9): 1172-1177. | Reference was not yet considered in previous guideline |
| Kaplan, L. J., et al. (1997). "Improved emergency department efficiency with a three-tier trauma triage system." Injury 28(7): 449-453. | Reference was not yet considered in previous guideline |
| Kappel, D. A., et al. (2011). "Does the rural trauma team development course shorten the interval from trauma patient arrival to decision to transfer?" Journal of Trauma-Injury Infection & Critical Care 70(2): 315-319. | Study type |
| Khetarpal, S., et al. (1999). "Trauma faculty and trauma team activation: impact on trauma system function and patient outcome." Journal of Trauma-Injury Infection & Critical Care 47(3): 576-581. | Reference was not yet considered in previous guideline |
| Klepner, S., et al. (2018). "Being Narrow Minded Is Not Always Bad: Focusing on Emergent Interventions in Undertriage Initiatives Improves Mortality Prediction." American Surgeon 84(8): 1277-1283. | Study can be assigned to a specific recommendation or predefined question (according to PICO scheme) |
| Knudson, P., et al. (1988). "Improving the field triage of major trauma victims." Journal of Trauma-Injury Infection & Critical Care 28(5): 602-606. | Reference was not yet considered in previous guideline |
| Kohn, M. A., et al. (2004). "Trauma team activation criteria as predictors of patient disposition from the emergency department." Academic Emergency Medicine 11(1): 1-9. | Reference was not yet considered in previous guideline |
| Kong, G., et al. (2015). "Current state of trauma care in China, tools to predict death and ICU admission after arrival to hospital." Injury 46(9): 1784-1789. | Study type |
| Kristiansen, L. H., et al. (2020). "Trauma team training at a "high-risk, low-incidence" hospital." Danish Medical Journal 67(3). | Study type |
| Kuhne, C. A., et al. (2004). "[Personnel and structural requirements for the shock trauma room management of multiple trauma. A systematic review of the literature]." Unfallchirurg 107(10): 851-861. | Reference was not yet considered in previous guideline |
| Kulkarni, S. S., et al. (2019). "Does Preexisting Practice Modify How Video Games Recalibrate Physician Heuristics in Trauma Triage?" Journal of Surgical Research 242: 55-61. | Study can be assigned to a specific recommendation or predefined question (according to PICO scheme) |
| Lee, D. B. and D. Battle (2018). "A Tiered Approach to Trauma Education in the Emergency Department." Journal of Trauma Nursing 25(5): 318-322. | Study type |
| Lehmann, R., et al. (2009). "The impact of advanced age on trauma triage decisions and outcomes: a statewide analysis." American Journal of Surgery 197(5): 571-574; discussion 574-575. | Study can be assigned to a specific recommendation or predefined question (according to PICO scheme) |
| Lossius, H. M., et al. (2000). "Efficiency of activation of the trauma team in a Norwegian trauma referral centre." European Journal of Surgery 166(10): 760-764. | Reference was not yet considered in previous guideline |
| Maluso, P., et al. (2016). "Trauma team size and task performance in adult trauma resuscitations." Journal of Surgical Research 204(1): 176-182. | Study type |
| Marr, M., et al. (2012). "Team play in surgical education: a simulation-based study." Journal of Surgical Education 69(1): 63-69. | Study type |
| Montgomery, K., et al. (2015). "Pharmacist's impact on acute pain management during trauma resuscitation." Journal of Trauma Nursing 22(2): 87-90. | Study type |
| Morris, R., et al. (2021). "Need for Emergent Intervention within 6 Hours: A Novel Prediction Model for Hospital Trauma Triage." Prehospital Emergency Care: 1-10. | Study can be assigned to a specific recommendation or predefined question (according to PICO scheme) |
| Morris, R. S., et al. (2020). "Redefining the Trauma Triage Matrix: The Role of Emergent Interventions." Journal of Surgical Research 251: 195-201. | Study can be assigned to a specific recommendation or predefined question (according to PICO scheme) |
| Muhm, M., et al. (2011). "[Preclinical prediction of prehospital injury severity by emergency physicians : approach to evaluate validity]." Anaesthesist 60(6): 534-540. | Study population |
| Norwood, S. H., et al. (2002). "A prehospital glasgow coma scale score < or = 14 accurately predicts the need for full trauma team activation and patient hospitalization after motor vehicle collisions." Journal of Trauma-Injury Infection & Critical Care 5 | Reference was not yet considered in previous guideline |
| Paige, J., et al. (2019). "Moving Along: Team Training for Emergency Room Trauma Transfers (T<sup>2</sup>ERT<sup>2</sup>)." Journal of Surgical Education 76(5): 1402-1412. | Study type |
| Peckler, B., et al. (2012). "Teamwork in the trauma room evaluation of a multimodal team training program." Journal of Emergencies Trauma & Shock 5(1): 23-27. | Study type |
| Rainer, T. H., et al. (2007). "Do trauma teams make a difference? A single centre registry study." Resuscitation 73(3): 374-381. | Study type |
| Rehn, M., et al. (2012). "Efficacy of a two-tiered trauma team activation protocol in a Norwegian trauma centre." British Journal of Surgery 99(2): 199-208. | Study type |
| Rice, Y., et al. (2016). "Implementation and Evaluation of a Team Simulation Training Program." Journal of Trauma Nursing 23(5): 298-303. | Study type |
| Rogers, A., et al. (2012). "Old and undertriaged: a lethal combination." American Surgeon 78(6): 711-715. | Study type |
| Rogers, A., et al. (2013). "Increased mortality with undertriaged patients in a mature trauma center with an aggressive trauma team activation system." European Journal of Trauma & Emergency Surgery 39(6): 599-603. | Study type |
| Rosqvist, E., et al. (2021). "Costs of hospital trauma team simulation training: a prospective cohort study." BMJ Open 11(6): e046845. | Study type |
| Ryan, J. M., et al. (1998). "Implementation of a two-tier trauma response." Injury 29(9): 677-683. | Reference was not yet considered in previous guideline |
| Ryb, G. E., et al. (2012). "Delayed trauma team activation: patient characteristics and outcomes." The Journal of Trauma and Acute Care Surgery 73(3): 695-698. | Study type |
| Schellenberg, M., et al. (2019). "Undertriaged trauma patients: Who are we missing?" The Journal of Trauma and Acute Care Surgery 87(4): 865-869. | Study type |
| Schweigkofler, U., et al. (2020). "[Emergency room activation due to trauma mechanism]." Unfallchirurg 123(5): 386-394. | Study type |
| Simon, B. J., et al. (1994). "Vehicular trauma triage by mechanism: avoidance of the unproductive evaluation." Journal of Trauma-Injury Infection & Critical Care 37(4): 645-649. | Reference was not yet considered in previous guideline |
| Smith, C. M., et al. (2011). "Major trauma CT scanning: the experience of a regional trauma centre in the UK." Emergency Medicine Journal 28(5): 378-382. | Study can be assigned to a specific recommendation or predefined question (according to PICO scheme) |
| Steele, R., et al. (2007). "Do the American College of Surgeons' "major resuscitation" trauma triage criteria predict emergency operative management?" Annals of Emergency Medicine 50(1): 1-6. | Reference was not yet considered in previous guideline |
| Svirsky, I., et al. (2013). "Resident-initiated advanced triage effect on emergency department patient flow." Journal of Emergency Medicine 45(5): 746-751. | Study population |
| Synnot, A., et al. (2017). "Prehospital notification for major trauma patients requiring emergency hospital transport: A systematic review." Journal of Evidence-based Medicine 10(3): 212-221. | Study type |
| Terregino, C. A., et al. (1997). "Secondary emergency department triage (supertriage) and trauma team activation: effects on resource utilization and patient care." Journal of Trauma-Injury Infection & Critical Care 43(1): 61-64. | Reference was not yet considered in previous guideline |
| Thompson, C. T., et al. (1992). "Community hospital level II trauma center outcome." Journal of Trauma-Injury Infection & Critical Care 32(3): 336-341; discussion 341-333. | Reference was not yet considered in previous guideline |
| Tinkoff, G. H. and R. E. O'Connor (2002). "Validation of new trauma triage rules for trauma attending response to the emergency department." Journal of Trauma-Injury Infection & Critical Care 52(6): 1153-1158; discussion 1158-1159. | Reference was not yet considered in previous guideline |
| Tominaga, G. T., et al. (2017). "Trauma resource designation: an innovative approach to improving trauma system overtriage." Trauma Surgery & Acute Care Open 2(1): e000102. | Study type |
| Torun, G. and V. A. Durak (2019). "The predictive value of triage early Warning Score (TEWS) on mortality of trauma patients presenting to the Emergency Department." Annali Italiani di Chirurgia 90: 152-156. | Study type |
| Vinjevoll, O. P., et al. (2018). "Evaluating the ability of a trauma team activation tool to identify severe injury: a multicentre cohort study." Scandinavian Journal of Trauma, Resuscitation & Emergency Medicine 26(1): 63. | Study type |
| Walcher, F., et al. (2013). "[TEAM R-G (Trauma Evaluation and Management Germany). Serves as a basis for an interdisciplinary training in the emergency room]." Unfallchirurg 116(7): 602-609. | Study type |
| Waldeck, S., et al. (2019). "[SMAR<sup>3</sup>T©-a new time-saving diagnostic emergency room management algorithm]." Chirurg 90(10): 845-850. | Study can be assigned to a specific recommendation or predefined question (according to PICO scheme) |
| Waydhas, C., et al. (2020). "ISS alone, is not sufficient to correctly assign patients post hoc to trauma team requirement." European Journal of Trauma & Emergency Surgery 16: 16. | Study can be assigned to a specific recommendation or predefined question (according to PICO scheme) |
| Wentling, J., et al. (2019). "Predictive Value of Point-of-care Lactate Measurement in Patients Meeting Level II and III Trauma Team Activation Criteria that Present to the Emergency Department: A Prospective Study." Journal of Emergencies Trauma & Shock | Study can be assigned to a specific recommendation or predefined question (according to PICO scheme) |
| Wisborg, T., et al. (2006). "Training multiprofessional trauma teams in Norwegian hospitals using simple and low cost local simulations." Education for Health 19(1): 85-95. | Study type |
| Wurmb, T., et al. (2005). "[The Wurzburg polytrauma algorithm. Concept and first results of a sliding-gantry-based computer tomography diagnostic system]." Anaesthesist 54(8): 763-768; 770-762. | Study type |
| Wuthisuthimethawee, P. (2017). "Trauma team activation criteria in managing trauma patients at an emergency room in Thailand." European Journal of Trauma & Emergency Surgery 43(1): 53-57. | Study type |
| Wuthisuthimethawee, P., et al. (2015). "Sustainable Effectiveness of Applying Trauma Team Activation in Managing Trauma Patients in the Emergency Department." Journal of the Medical Association of Thailand 98(9): 847-851. | Study type |
| Zeindler, M., et al. (2020). "Comparative analysis of MGAP, GAP, and RISC2 as predictors of patient outcome and emergency interventional need in emergency room treatment of the injured." European Journal of Trauma & Emergency Surgery 13: 13. | Study type |

# S4 Evidence Table

##### Criteria for trauma team activation

| **Study: Reference, aim, design, setting** | **Participants: selection criteria, characteristics** | **N Participants;  Activation criteria** | **Main outcomes** | **Assessment: LoE, risk of bias; Conclusions** |
| --- | --- | --- | --- | --- |
| **Bieler (2021)**  “Evaluation of a standardized instrument for post hoc analysis of trauma‑team‑activation‑criteria in 75,613 injured patients an analysis of the TraumaRegister DGU®”. *Eur J Trauma Emerg Surg*, 1-9.  **Study design**  Prognostic cross-sectional study  (TraumaRegister DGU®)  **Aim of the study**  „to examine whether the catalogue can identify severely injured patients with an increased mortality risk to evaluate in the future especially with regard to the positive predictive value of new and existing activation criteria for trauma teams.“  **Setting**  Germany, 2007-2016 | **Inclusion criteria**   - Adults (age ≥16) - Maximum AIS ≥2   **Exclusion criteria**   - Patients transferred in as well as patients transferred out within 48 h   **Characteristics**  n.r. | **Participants**  N=75,613 patients  **(Potential) activation criteria**  1: Cardiopulmonary resuscitation (N=3162)  2: Insertion of a chest tube (N=8823)  3: Administration of catecholamine (N=13,150)  4: Drop in GCS ≥2 points (N=3706)  5: Saturation of peripheral oxygen<90% (N=9484)  6: Hypothermia <35 °C (N=3040)  7: Shock index >0.9 (N=17,720)  8: Respiratory rate <9 or >29/min (N=3207)  9: Advanced Airway (N=22771)  10: GCS score <9 (N=15099)  11: SBP <90 mmHg (N=11212) | **Mortality, n/N (%)**  1: 2409/3162 (76.2)  2: 2033/8823 (23.0)  3: 4692/13,150 (35.7)  4: 477/3706 (12.9)  5: 2989/9484 (31.5)  6: 880/3040 (28.9)  7: 3165/17,720 (17.9)  8: 1452/3207 (45.3)  9: 6154/22771 (27.0)  10: 5660/15099 (37.5)  11: 3322/11212 (29.6)  **Mortality when only a single criterion was fulfilled, n/N (%)**  1: 0/14 (0.0)  2: 0/263 (0.0)  3: 0/94 (0.0)  4: 6/420 (1.4)  5: 7/514 (1.4)  6: 1/88 (1.1)  7: 3/1639 (0.2)  8: 1/45 (2.2)  9: 3/592 (0.5)  10: 0/166 (0.0)  11: 0/186 (0.0) | **Level of evidence**  2b  **Risk of bias**  no tool available for prognostic studies  **Authors’ conclusion**  n.r. for comparison of interest  **Reviewers’ conclusion**  The primary aim of the study was to evaluate a post hoc criteria catalogue for trauma-team-activation and not to examine the criteria catalogue as activation criteria themselves. According to the authors data availability was unsatisfactory for temperature and respiratory rate. No patient characteristics were reported for the overall population. |
| **Brown (2016)**  “Systolic blood pressure criteria in the National Trauma Triage Protocol for geriatric trauma: 110 is the new 90”. *J Trauma Acute Care Surg* 2015; 78(2):352-359.  **Study design**  Prognostic cross-sectional study  (National Trauma Data Bank)  **Aim of the study**  “to evaluate the impact of substituting an SBP of less than 110 mm Hg for the current SBP of less than 90 mm Hg criterion within the NTTP on triage performance and mortality.”  **Setting**  USA, 2010-2012 | **Inclusion criteria**   - age >15 y - transported from the scene of injury   **Exclusion criteria**   - interfacility transfer - death on arrival   **Characteristics (for different age groups)**  Age [y], median (IQR)  Geriatric: 80 (73–86) Adult: 37 (25–50), p<0.01  Male gender, %  Geriatric: 39 Adult: 71, p<0.01  Blunt injury, %  Geriatric: 99 Adult: 85, p<0.01  Prehospital SBP [mmHg], median (IQR)  Geriatric: 144 (128–164) Adult: 131 (118–146), p<0.01  ISS, median (IQR)  Geriatric: 9 (4–10) Adult: 6 (4–13), p<0.01 | **Participants**  N=1,555,944 patients overall;  N=438,828 geriatric cohort (age >65 y); N=1,117,116 adult cohort (age 16-65 y)  **(Potential) activation criteria**  1: SBP <110 mmHg  2: SBP <90 mmHg  3: presence of physiologic Step 1 NTTP criteria (GCS score ≤13, SBP <90 mm Hg, respiratory rate (RR) <10 or RR >29) or presence of anatomic Step 2 NTTP criteria (penetrating injury, flail chest, open skull fracture, ≥2 proximal long bone fractures, pelvic fracture, crush injury, amputation, paralysis) using SBP <110 mmHg  4: presence of physiologic Step 1 NTTP criteria (GCS score ≤13, SBP <90 mm Hg, respiratory rate (RR) <10 or RR >29) or presence of anatomic Step 2 NTTP criteria (penetrating injury, flail chest, open skull fracture, ≥2 proximal long bone fractures, pelvic fracture, crush injury, amputation, paralysis) using SBP <90 mmHg  **Variables included in logistic regression to calculate ROC**   - Sex - Race - Mechanism - Pre-hospital time - Transportation mode - prehospital and admission vital signs - ISS - urgent surgery - ICU admission - trauma center level | **Results for primary outcome (trauma center need)***  Sensitivity for geriatrics, %  1: 13 2: 5 3: 44 4: 40  Sensitivity for adults, %  1: 23 2: 10 3: 67 4: 62  Specificity for geriatrics, %  1: 93 2: 99 3: 71 4: 75  Specificity for adults, %  1: 90 2: 98 3: 62 4: 67  Positive predictive value for geriatrics, %  1: 50 2: 66 3: 44 4: 45  Positive predictive value for adults, %  1: 63 2: 79 3: 58 4: 59  Negative predictive value for geriatrics, %  1: 68 2: 67 3: 71 4: 71  Negative predictive value for adults, %  1: 61 2: 59 3: 70 4: 69  ROC AUC (95% CI), AIC for geriatrics  1: 0.532 (0.530 to 0.534), 364865 2: 0.519(0.517 to 0.522), 365258 3: 0.575 (0.572 to 0.577), 361316 4: 0.574 (0.571 to 0.576), 363319  ROC AUC (95% CI), AIC for adults  1: 0.564 (0.563 to 0.566), 980176 2: 0.539 (0.538 to 0.541), 979913 3: 0.641 (0.640 to 0.642), 1022308 4: 0.646 (0.645 to 0.647), 1013075  Undertriage** reduction by substituting an SBP <110 mmHg, %  Geriatric: 4.4 Adult: 4.3  **defined as a subject who did not have any Step 1 or Step 2 NTTP criteria present but met the definition of TCN  Overtriage*** increase by substituting an SBP <110 mmHg, %  Geriatric: 4.3 Adult: 5.3  ***defined as a subject who did have at least one Step 1 or Step 2 NTTP criteria present but did not meet the definition of TCN  Optimal SBP for geriatrics (<122 mmHg), sensitivity [%], specificity [%]  22, 83  Optimal SBP for adults (<118 mmHg), sensitivity [%], specificity [%]  32, 73  *trauma center need defined as a composite of ISS <15, ICU admission ≥24 h, need for urgent surgery (defined as ED disposition to the operating room), or death in the ED  **Results for secondary outcome (mortality)**  Optimal SBP for geriatrics (<118 mmHg), sensitivity [%], specificity [%]  29, 86  Optimal SBP for adults (<106 mmHg), sensitivity [%], specificity [%]  49, 88  Mortality of patients newly triaged because of substituting SBP value compared to regular SBP, AOR (95% CI); model c statistics (95% CI)  Geriatric: 1.03 (0.88–1.20), p=0.76; 0.910 (0.904–0.915) | **Level of evidence**  2b  **Risk of bias**  no tool available for prognostic studies  **Authors’ conclusion**  “Substituting an SBP of less than 110 mm Hg criterion in geriatric patients results in discrimination as good as the current SBP of less than 90 mm Hg criterion, with superior improvements in undertriage relative to overtriage. Geriatric patients who would be newly triaged positive under this change have a risk of mortality similar to those under the current SBP triage criterion, warranting transport to a trauma center.”  **Reviewers’ conclusion**  A large amount of data was missing. Authors used multiple imputation methods, but state that the amount of missing data was larger than suitable to avoid risk of bias. However, sensitivity analyses for complete data and best-case scenarios revealed comparable results.  Only data for the first two steps in activation were provided in the database excluding trauma activations for mechanism of injury (step 3) and special considerations (step 4). Furthermore, the authors state that it is possible that a group of patients with no other activation criteria who would have been captured by the SBP of less than 110 mm Hg criterion were not taken to a trauma center under the current SBP of less than 90 mm Hg criterion and thus would not be represented in the database. If this group is substantial and ultimately does not require trauma center care, a significantly greater increase in overtriage from that reported here is possible, with the attendant problems highlighted earlier. The authors could not account for this scenario.  In this study, the overtriage rate represents the false positive patients and the undertriage rate represents the false negative patients. |
| **Cull (2019)**  “Development of Trauma Level Prediction Models Using Emergency Medical Service Vital Signs to Reduce Over- and Undertriage Rates in Penetrating Wounds and Falls of the Elderly”. *Am Surg* 2019; 85(5): 524-529.  **Study design**  Prognostic cross-sectional study  (National Trauma Data Bank)  **Aim of the study**  “analyzes the influence of EMS vital signs on injury severity and triage level for use in developing a predictive model when assessing GF [geriatric falls], GSW [gunshot wounds], and SW [stab wound] patients.”  **Setting**  USA, 2013-2015 | **Inclusion criteria**  for geriatric fall:   - blunt trauma - E-code fall on the same level - other - age ≥65 y - transported via ground ambulance - vitals sign type EMS   for firearm:   - penetrating injury - E-code all listed firearm mechanism - Age >0 y [data were filtered for patients >14 years] - Transported via ground ambulance - vitals sign type EMS   cut/pierce:   - penetrating injury - E-code all listed cut/pierce mechanism - Age >0 [data were filtered for patients >14 years] - Transported via ground ambulance - vitals sign type EMS   **Exclusion criteria**   - missing vital data - vital data not in required ranges (60≤SBP≤240; 0<PR≤200; 0<RR≤40; GCS≤15)   **Characteristics**  n.r. | **Participants**  N=157,164 patients  **(Potential) Activation criteria**  1: Falls (N=92780); equation for predictive model = 0.286 + 0.00267 x SBP – 0.00538 x PR + 0.00656 x RR + 0.0444 x GCS + 0.000006 x SBP^2^ – 0.003209 x GCS^2^ – 0.000297 x SBP x GCS + 0.000357 x PR x GCS – 0.000593 x RR x GCS  2: Gunshot wounds (N=32440); equation for predictive model= 0.861 – 0.00134 x SBP + 0.00075 x PR – 0.02778 x RR + 0.0844 x GCS + 0.000020 x SBP^2^ + 0.000014 x PR^2^ + 0.000583 x RR^2^ – 0.006277 x GCS^2^ – 0.000035 x SBP x PR + 0.000083 x SBP x RR – 0.000282 x SBP x GCS + 0.000108 x PR x GCS  3: Stab wounds (N=31944); equation for predictive model= 0.587 – 0.00276 x SBP – 0.00128 x PR + 0.01023 x RR – 0.02529 x GCS + 0.000018 x SBP^2^ + 0.000024 x PR^2^ + 0.000255 x RR^2^ – 0.000015 x SBP x PR – 0.000064 x SBP x RR – 0.000078 x PR x RR | Overtriage*, n/N (%)  1: 34418/92780 (37.10) 2: 14512/32440 (44.73) 3: 6885/31944 (21.55)  *coded trauma level indicated no trauma activation, but predictive model indicated activation  Undertriage**, n/N (%)  1: 3245/92780 (3.50) 2: 1191/32440 (3.67) 3: 1171/31944 (3.67)  ** coded trauma level indicated trauma activation, but predictive model indicated no activation  Accuracy***, n/N (%)  1: 55117/92780 (59.41) 2: 16737/32440 (51.59) 3: 23888/31944 (74.78)  ***coded trauma level matches predictive model | **Level of evidence**  2b  **Risk of bias**  no tool available for prognostic studies  **Authors’ conclusion**  “Our developed trauma level prediction models enable health providers to predict trauma activation levels that result in OT and UT rates within the recommended ranges by the Committee on Trauma.”  **Reviewers’ conclusion**  The cribari method was used to calculate over-and undertriage, but there are concerns regarding the validity of this method for calculation of undertriage. Over- und undertriage are calculated solely based on ISS. No patient characteristics were reported. |
| **Damme (2016)**  “Isolated prehospital hypotension correlates with injury severity and outcomes in patients with trauma”. *Trauma Surg Acute Care Open* 2016; 1(1): e000013.  **Study design**  Prognostic cross-sectional study  **Aim of the study**  “to determine whether isolated prehospital hypotension portends poor outcomes and correlates with injury severity”  **Setting**  USA, 2014 | **Inclusion criteria**  n.r.  **Exclusion criteria**   - Patients who remained hypotensive on admission - Who were transferred by a non-EMS service and thus had no prehospital SBP documentation - With incomplete data   **Characteristics**  Male gender, %  hypotension: 59  normotension: 60, p=0.77  Age [y], mean ± standard error  hypotension: 38.8±2.7  normotension: 51.1±1.6, p<0.0001  Mechanism of injury (%)  Blunt:  hypotension: 90  normotension: 92  Burn:  hypotension: 1  normotension: 1  Penetrating:  hypotension: 9  normotension: 7, p overall=0.7593  SBP [mmHg], mean ± standard error  Pre-hospital:  hypotension: 99.11±1.75  normotension: 143.44±1.59, p<0.0001  ED:  hypotension: 132.65±1.39  normotension: 148.47±1.69, p<0.0001  GCS, mean± standard error  Pre-hospital:  hypotension: 12.81±0.44  normotension: 14.38±0.13, p<0.0001  ED:  hypotension: 12.78±0.47  normotension: 14.37±0.14, p<0.0001 | **Participants**  N=287 patients  **(Potential) activation criteria**  Hypotension defined as SBP ≤110 mm Hg (n=81 patients)  Normotension defined as SBP >110 mm Hg (n=206 patients) | ICU admission, %  hypotension: 56.79  normotension: 22.82, p<0.0001  ICU LOS [d], mean ± standard error  hypotension: 3.23±0.71  normotension: 0.71±0.17, p<0.0001  Ventilator days, mean ± standard error  hypotension: 3.38±1.20  normotension: 0.27±0.08, p=0.0001  Packed red blood cells [first 24 h], %  hypotension: 22±8  normotension: 6±2, p=0.0114  CAVE: It is uncertain, whether data for PRBC reflect proportions or mean (SE)  ISS, mean± standard error  hypotension: 12.27±1.12  normotension: 9.22±0.49, p<0.0001 | **Level of evidence**  2b  **Risk of bias**  no tool available for prognostic studies  **Authors’ conclusion**  “Isolated prehospital hypotension in patients in the trauma and emergency department correlates with increased injury severity and portends worse outcomes despite a normal blood pressure reading at admission. Prehospital hypotension must be given heavy consideration in triage, as these patients may be transiently hypotensive and appear less critical than their true status.”  **Reviewers’ conclusion**  This was a prospective single center study. Information on the study’s inclusion criteria are missing. Both patient groups differed significantly in age and pre-hospital and ED-GCS. |
| **Dehli (2016)**  "Evaluation of a trauma team activation protocol revision: a prospective cohort study". *Scandinavian Journal of Trauma, Resuscitation and Emergency Medicine* 2016; 24(1): 105.  **Study design**  Prognostic cross-sectional study  **Aim of the study**  “we evaluated the protocol revision by comparing over- and undertriage in the former and present set of criteria”  **Setting**  Norway, 2013-2014 | **Inclusion criteria**   - severely injured patients (ISS >15) with our without a TTA - patients admitted with TTA   **Exclusion criteria**   - patients transferred >24 h after injury   In the following, only data since the implementation of the new protocol were extracted  **Characteristics (overall)**  Male gender, n (%)  226 (69.8%)  Age [y[, mean (range)  41 (0–101)  ISS, median (IQR)  10 (2, 20)  Predominant mechanism of injury (%)  Penetrating 3.4% Blunt 96.6% | **Participants**  N=324 patients  **(Potential) activation criteria**  Vital parameters:  1. Airway obstruction, stridor N=4  2. Tachypnoe (adults, respiratory rate >30/min) N=14  3. Heart rate >130 (adults) N=3  4. Systolic BP <90 mmHg N=9  5. Lowered level of consciousness (GCS score <13) N=87  Extent of injuries:  6. Flail chest N=2  7. Unstable fracture of the pelvis. Fracture in two or more long bones N=5  8. Traumatic amputation or crush injury above wrist/ankle N=1  9. Injury in two or more body regions (head/neck/chest/abdomen/pelvis/ femur/back) N=61  10. Paralysis N=10  11. Penetrating injury of the head/neck/chest/abdomen/pelvis/ groin/back N=5  12. 2. or 3. degree burn injury >15 % body surface (children >10 %) N=5  13. Burn injury with inhalation injury N=5  14. Hypothermia (core temperature <32 °C) N=11  Mechanism of injury  15. Ejected from vehicle N=6  16. Co-passenger dead N=5  17. Trapped in wreck N=9  18. Pedestrian or cyclist hit by motor vehicle N=15  19. Fall from >5 m N=20  20. Avalanche accident N=1  Unknown criteria:  21. Estimated time from arrival<15 min N=8  22. Trauma team leader requested TTA N=5  23. Anesthesiologist in ambulance helicopter requested TTA N=6  24. Unknown/undocumented reason for TTA N=20 | **Accuracy, n/N (%)** fraction of patients correctly classified as severely injured (ISS >15), with N=all patients meeting the activation criterion  1: 2/4 (50)  2: 10/14 (71)  3: 0/3 (0)  4: 5/9 (56)  5: 33/87 (38)  6: 1/2 (50)  7: 2/5 (40)  8: 0/1 (0)  9: 9/61 (15)  10: 8/10 (80)  11: 0/5 (0)  12: 2/5 (40)  13: 2/5 (40)  14: 3/11 (27)  15: 4/6 (67)  16: 2/5 (40)  17: 3/9 (33)  18: 2/15 (13)  19: 10/20 (50)  20: 0/1 (0)  21: 0/8 (0)  22: 0/5 (0)  23: 0/6 (0)  24: 0/20 (0)  fraction of patients correctly classified as severely injured (need for emergency procedure*), with N=all patients meeting the activation criterion  1: 3/4 (75)  2: 3/14 (21)  3: 2/3 (67)  4: 4/9 (44)  5: 28/87 (32)  6: 0/2 (0)  7: 0/5 (0)  8: 1/1 (100)  9: 8/61 (13)  10: 1/10 (10)  11: 3/5 (60)  12: 3/5 (60)  13: 2/5 (40)  14: 2/11 (18)  15: 0/6 (0)  16: 0/5 (0)  17: 1/9 (11)  18: 2/15 (13)  19: 3/20 (15)  20: 0/1 (0)  21: 0/8 (0)  22: 0/5 (0)  23: 0/6 (0)  24: 0/20 (0)  *emergency procedures were endotracheal intubation, damage control thoracotomy, damage control laparotomy, extraperitoneal pelvic packing, intervention radiology, craniotomy, insertion of intracranial pressure bolt, chest tube insertion, external fracture stabilization, other procedures to stabilize airways, respiration or circulation | **Level of evidence**  3b↓  **Risk of bias**  no tool available for prognostic studies  **Authors’ conclusion**  “The low number of patients makes it difficult to draw conclusions about individual criteria for TTA, and even though some criteria were seldom used, the proportion of correctly triaged patients was high. However, based on the findings in the present study, we suggest that further changes in the present TTA criteria will not help to reduce overtriage. Another way to limit unnecessary use of limited resources may be to introduce a two-tiered TTA, with a low threshold for mobilizing a smaller team based on MOI information from the prehospital services, and the full team only when alarming vital signs or anatomical injuries are reported.”  **Reviewers’ conclusion**  This was a prospective single center study. The study was downgraded, because it was underpowered to show a difference for the individual activation criteria. The low number of patients should be kept in mind when interpreting the results for individual TTA criteria. Furthermore, not all criteria are assessed for every admission since there is no difference if one or more criteria are fulfilled. |
| **Guyette (2015)**  “A comparison of prehospital lactate and systolic blood pressure for predicting the need for resuscitative care in trauma transported by ground”. *J Trauma Acute Care Surg* 2015; 78(3): 600-606.  **Study design**  Prognostic cross-sectional study  **Aim of the study**  “to compare prehospital point-of-care lactate (P-LAC) with systolic blood pressure (SBP) for predicting the need for resuscitative care (RC) in trauma patients transported by ground emergency medical services”  **Setting**  North America, 2011-2012 | **Inclusion criteria**   - Prehospital SBP ≤100 mm Hg - Blunt or penetrating trauma - Transported by emergency medical services to a Level I or II trauma center - Valid lactate measurement   **Exclusion criteria**   - age <15 years - obvious isolated penetrating head injury - drowning - asphyxia caused by hanging - burns on >20% of total body surface area - prisoner status - SBP ≤70 mm Hg   **Characteristics**  n.r. for comparison of interest | **Participants**  N=387 patients  **(Potential) activation criteria**   - Point-of-care lactate - SBP - Shock index (heart rate/SBP) - GCS score - Any airway/bag valve mask attempted   **Variables included in multivariable logistic regression**   - Age - Sex - Mechanism of injury - Prehospital vital signs (SBP, SI, GCS score) - Airway status - And regional site | **Adjusted outcomes**  Logistic regression analysis for need for resuscitative care*, OR (95% CI)  Point-of-care lactate**:  1 mmol/L difference in point-of-care lactate within the range of <2.5: 1.76 (0.41-12.93)  1 mmol/L difference in point-of-care lactate within the range of 2.5-3.9: 3.61 (1.67-8.35)  1 mmol/L difference in point-of-care lactate within the range of ≥4.0: 0.97 (0.87-1.07)  SBP (per 5 mm Hg): 0.92 (0.73-1.15)  Shock index (per increment of 0.1): 1.21 (1.06-1.38)  Initial GCS score (per increment of 1): 1.01 (0.90-1.13)  Any airway/bag valve mask attempted: 4.55 (1.40-15.43)  **Unadjusted outcomes**  **Accuracy, n/N (%)** fraction of patients correctly classified as severely injured (need for resuscitative care), with N=all patient meeting the activation criterion:  Point-of-Care-Lactate ≥2.5 mmol/L: 65/228 (28.5)  SBP ≤90 mm Hg: 47/213 (22.1)  Point-of-Care-Lactate ≥2.5 mmol/L and SBP 91-100 mm Hg: 21/93 (22.6)  Sensitivity for detection of patients with need of resuscitative care*, % (95% CI)  Point-of-care lactate ≥2.5mmol/L: 93 (84-98)  SBP ≤90 mm Hg 67 (55-78), p<0.001 for difference  Negative predictive value for detection of patients with need of resuscitative care*, % (95% CI)  Point-of-care lactate <2.5mmol/L: 97(93-99)  SBP >90 mm Hg 87 (81-91)  Area under the receiver operating characteristic curve, % (95% CI)  Point-of-care lactate 0.78 (0.73-0.83), statistically significant superior to  SBP 0.59 (0.53-0.66) and  Shock index 0.66 (0.60-0.74)  *need for resuscitative care defined as any of the following within 6 hours of emergency department arrival:   - blood transfusion of 5 U or greater - intervention for haemorrhage including thoracotomy, laparotomy, pelvic fixation, or interventional radiology embolization - death (including death before hospital arrival)   **Modeled as linear spline with knots at 2.5 mmol/L and 4.0 mmol/L. The estimate for a given lactate range is the ratio of odds for the need for resuscitative care between two patients who both have lactate levels within the same range (e.g., 2.5-3.9) and have the same covariate data except that their lactate levels differ by 1 mmol/L. | **Level of evidence**  2b  **Risk of bias**  no tool available for prognostic studies  **Authors’ conclusion**  “P-LAC obtained at the scene of injury is strongly associated with the need for RC. P-LAC is superior to other early surrogates for hypoperfusion (SBP and SI) in predicting the need for RC in trauma patients with 70 mm Hg G SBP e 100 mm Hg.”  **Reviewers’ conclusion**  There was a large proportion of patients for whom no (valid) lactate measure could be performed. Emergency service personnel as well as receiving personnel in the hospital was blinded to the point-of-care lactate value. Sensitivity analyses using two alternative definitions of resuscitative care found no differences in results. The authors state that the cutoff points are not intended to be clinical decision points and may result in overtriage if they are extended to a patient population that does not meet their eligibility criteria. |
| **Hasler (2011)**  “Systolic blood pressure below 110 mmHg is associated with increased mortality in blunt major trauma patients: Multicentre cohort study”. *Resuscitation* 2011; 82(9): 1202-7.  **Study design**  Prognostic cross-sectional study  (Trauma Audit and Research Network)  **Aim of the study**  “to examine the association of SBP with mortality in blunt trauma patients.”  **Setting**  Europe, 2000-2009 | **Inclusion criteria**   - Injured patients - Adults ≥16 y   **Exclusion criteria**   - penetrating injuries - concomitant head trauma (AIS 3+) - transferals from a non-TARN hospital - referrals to a non-TARN hospital - missing data for SBP or GCS   **Characteristics (overall)**  Age [y], median (IQR)  51.1 (32.8 to 67.4)  Male gender, n (%)  28,694 (60)  ISS, median (IQR)  9 (8 to 10)  GCS, median (IQR)  15 (15 to 15)  SBP, median (IQR)  135 (120 to 152) | **Participants**  N=47,927 patients  **(Potential) activation criteria**  SBP at ED arrival <70 mmHg (N=346)  SBP at ED arrival 70–79 mmHg (N=327)  SBP at ED arrival 80–89 mmHg (N=761)  SBP at ED arrival 90–99 mmHg (N=1584)  SBP at ED arrival 100–109 mmHg (N=3191)  SBP at ED arrival 110–119 mmHg (N=5380)  SBP at ED arrival 120–129 mmHg (N=7393)  SBP at ED arrival 130–139 mmHg (N=7971)  SBP at ED arrival 140–149 mmHg (N=7347)  SBP at ED arrival 150–159 mmHg (N=5394)  SBP at ED arrival 160–169 mmHg (N=3258)  SBP at ED arrival 170–179 mmHg (N=1953)  SBP at ED arrival 180–189 mmHg (N=1299)  SBP at ED arrival 190–199 mmHg (N=816)  SBP at ED arrival ≥200 mmHg (N=907)  **Adjustment criteria in multivariate analysis using mixed effect logistic regression**   - age - gender - ISS - GCS | Multivariate analysis for mortality, OR (95% CI)  SBP <70 mmHg: 5.98 (4.42 to 8.09)  SBP <90 mmHg: 2.90 (2.20 to 3.84)  SBP <100 mmHg: 2.11 (1.65 to 2.70)  SBP <110 mmHg: 1.69 (1.35 to 2.12)  SBP <130 mmHg: reference category  ROC curve = 0.89 and AIC = 10811.15 | **Level of evidence**  2b  **Risk of bias**  no tool available for prognostic studies  **Authors’ conclusion**  “We recommend triaging adult blunt trauma patients with a SBP <110 mmHg to resuscitation areas within dedicated trauma units for close monitoring and appropriate management.”  **Reviewers’ conclusion**  This study included a high number of patients and performed several adjusted analyses for assessing the impact of SBP on mortality. The authors could not adjust for other possible confounders such as base excess, lactate levels, body temperature or co-morbidities, but the analysis showed a good model fit. SBP was measured using either automated or manual non-invasive blood pressure measurements, which might bias the results. Further, SBP measures were performed at arrival in the emergency department. When interpreting the results, one should keep in mind that the study population includes patients with severe traumatic injuries more frequently due to the definition of trauma according to the TARN registry. |
| **Hasler (2012)**  “Systolic blood pressure below 110 mmHg is associated with increased mortality in penetrating major trauma patients: Multicentre cohort study”. *Resuscitation* 2012; 83(4): 476-81.  **Study design**  Prognostic cross-sectional study  (Trauma Audit and Research Network)  **Aim of the study**  “to determine the association between different SBP cut-offs and overall mortality at 30 days after admission in patients with penetrating trauma.”  **Setting**  Europe, 2000-2009 | **Inclusion criteria**   - Adults ≥16 y - With penetrating trauma   **Exclusion criteria**   - blunt injuries - concomitant head trauma (AIS 3+) - transferals from a non-TARN hospital - referrals to a non-TARN hospital   **Patient characteristics (overall)**  Age [y], median (IQR)  30.0 (22.5 to 41.4)  Male gender, n (%)  2991 (86.9)  ISS, median (IQR)  9 (9 to 14)  GCS, median (IQR)  15 (15 to 15)  SBP, median (IQR)  126 (107 to 142) | **Participants**  N=3444 patients  **(Potential) activation criteria**  ED SBP <70 mmHg (N=153)  ED SBP 70–89 mmHg (N=276)  ED SBP 90–109 mmHg (N=520)  ED SBP 110–129 mmHg (N=941)  ED SBP 130–149 mmHg (N=946)  ED BP 150–169 mmHg (N=444)  ED SBP ≥170 mmHg (N=164)  **Adjustment criteria in multivariate analysis using mixed effect logistic regression**   - age - gender - ISS - GCS | Multivariate analysis for mortality [30d], OR (95% CI)  SBP <70 mmHg: 10.3 (4.76 to 22.2)  SBP 70–89 mmHg: 4.01 (2.02 to 7.95)  SBP 90–109 mmHg: 2.22 (1.09 to 4.50)  SBP 110–129 mmHg: 1.00 (reference)  SBP 130–149 mmHg: n.r.  SBP 150–169 mmHg: 0.65 (0.22 to 1.91)  SBP ≥170 mmHg: 0.20 (0.03 to 1.17),  p for linear trend <0.001  AUROC= 0.91 and AIC = 580 | **Level of evidence**  2b  **Risk of bias**  no tool available for prognostic studies  **Authors’ conclusion**  “We recommend that penetrating trauma patients with a SBP <110 mmHg are triaged to resuscitation areas within dedicated, appropriately specialised, high-level care trauma centres.”  **Reviewers’ conclusion**  The authors could not adjust for other possible confounders such as base excess, lactate levels, body temperature or co-morbidities. SBP was measured using either automated oscillometry or manual sphygmanometry, which might bias the results. SBP was measured on admission to ED. When interpreting the results, one should keep in mind that the study population includes patients with severe traumatic injuries more frequently due to the definition of trauma according to the TARN registry. |
| **Heindl (2021)**  “Emergency intervention rate in the emergency room depending on the alerting criteria. Prospective data analysis of a supraregional trauma center”. *Unfallchirurg* 2021; 4: 40.  **Study design**  Prognostic cross-sectional study  **Aim of the study**  “to describe ER patients according to the TTA criteria and to collect the corresponding emergency intervention rates in ER.“  **Setting**  Germany, 2017 | **Inclusion criteria**   - Patients who were primarily admitted by ground ambulance or air ambulance after an accident and were admitted via the shock room - Patients admitted to the shock room under resuscitation   **Exclusion criteria**   - Patients treated as self-referrals in the shock room without a previous accident or without treatment by the ambulance service - Patients referred secondarily after primary care at another clinic   **Characteristics**  Age [y], mean ± SD  A-criteria: 48.3± 28.8 B-criteria: 48.7± 22.9 Null criteria: 66± 19.7 p significant for comparison between A and Null and B and Null  ISS mean ± SD  A-criteria: 20.6± 21.3 B-criteria: 8.0± 7.2, p significant compared to A Null criteria: 5.6± 8.2, p significant compared to A  Admission GCS mean ± SD  A-criteria: 7.8± 5.5  B-criteria: 14.2± 1.8 p significant compared to A Null criteria: 13.8± 1.7 p significant compared to A  Systolic blood pressure at admission [mm Hg] mean ± SD  A-criteria: 99.7± 56.7 B-criteria 143.7± 25.4, p significant compared to A Null criteria: 141.7± 26.6, p significant compared to A | **Participants**  N=164 patients  **(Potential) activation criteria**  A-criteria (abnormal vital signs, obvious severe injury, prehospital intervention ) N=32  B-criteria (mechanism of injury) N=84  Null criteria (Criteria not mentioned in the criteria catalog of the S3 guideline; activations generally based on the assessment of the emergency physician alone) N=48 | Mortality (%)  A: 31.3 B: 2.4 Null: 2.1, p<0.001  Mechanism of injury - multiple injuries, (%)  A: 59 B: 67 Null: 29  Mechanism of injury - single injury, (%)  A: 41 B: 31 Null: 54  Mechanism of injury - no injury, (%)  A: 0 B: 2 Null: 17  Emergency intervention, (%)  A: 75 B: 6 Null: 2.1  Intubation, (%)  A: 71.9 B: 5.9 Null: 2  Chest tube, (%)  A: 34.4 B: 1.2 Null: 0  Cardiopulmonary resuscitation, (%)  A: 31.3 B: 0 Null: 0  Transfusion, (%)  A: 6.3 B: 0 Null: 0  Coagulation substitution, (%)  A: 15.6 B: 0 Null: 0  External pelvic stabilization, (%)  A: 9.4 B: 0 Null: 0  Surgical hemostasis, (%)  A: 0 B: 0 Null: 0 | **Level of evidence**  2b  **Risk of bias**  no tool available for prognostic studies  **Authors’ conclusion**  “Differentiation according to the TTA criteria results in patient collectives with different injury severity and emergency intervention rates. This result justifies considerations to adjust team composition based on TTA criteria, as long as it is ensured that critical conditions can be identified and remedied by adapted teams“  **Reviewers’ conclusion**  This was a prospective single center study. Except for the mortality rate there are no p values available for the outcome variables. Therefore, statements on the significance of these results are not possible. |
| **Hranjec (2012)**  “Mortality Factors in Geriatric Blunt Trauma Patients: Creation of a Highly Predictive Statistical Model for Mortality Using 50,765 Consecutive Elderly Trauma Admissions from the National Sample Project” *Am Surg*. 2012; 78(12): 1369–1375.  **Study design**  Prognostic cross-sectional study  (National Trauma Database)  **Aim of the study**  “to create a geriatric-specific model that would accurately predict in-hospital mortality in injured elderly trauma patients while adjusting for multiple covariates.”  **Setting**  USA, 2003-2006 | **Inclusion criteria**   - Age ≥65 - known outcome variable mortality/survival to discharge - hospital length of stay of at least 24 hours - ISS >0   **Exclusion criteria**  n.r.  **Patient characteristics (overall)**  Male gender, n (%)  24,603 (42.4) | **Participants**  N=57,973 patients  **(Potential) activation criteria**  1: Age  2: Gender  3: Motor GCS score  4: SBP  5: Temperature*  6: Mechanical ventilation  **Variables included in logistic regression**   - Age - Gender - ISS - Motor GCS score - SBP - Temperature - Presence of mechanical ventilation   * Even if the paper refers to degrees Celsius, it can be assumed from the values that it is degrees Fahrenheit | **Adjusted outcomes**  Logistic regression for prediction of mortality, OR (95% CI)  1: Age  >85y: 2 (1.8–2.2) 65–75 y: 1.86 (1.7–2.0) 65 y: reference  2: Gender  Female: 0.77 (0.7–0.8) Male: reference  3: motor GCS  1: 4.49 (4.0–5.0) 2–5: 2.82 (2.5–3.2) 6: reference  4: SBP  0–60 mmHg: 1.69 (1.1–2.7) 60–90 mmHg: 1.3 (1.1–1.6) 90-120 mmHg: reference 120–150 mmHg: 0.62 (0.6–0.7) 150–180 mmHg: 0.67 (0.6–0.8)  5: Temperature  65–97.7: 1.23 (1.1–1.4) 97.7–101.3: reference >101.3: 1.24 (0.5–2.5)  6: mechanical ventilation  Yes: 5.1 (4.6–5.6) No: reference | **Level of evidence**  2b  **Risk of bias**  no tool available for prognostic studies  **Authors’ conclusion**  “Clearly, a separate geriatric model for predicting outcomes is not only warranted, but necessary.”  **Reviewers’ conclusion**  Because of the high share of missing data on some items such as SBP and mechanical ventilator, an additional analysis of patients with complete data was performed, which revealed comparable results. Some important variables affecting mortality such as respiratory rate could not be included in the predictive model. |
| **Ichwan (2014)**  “Geriatric-Specific Triage Criteria Are More Sensitive Than Standard Adult Criteria in Identifying Need for Trauma Center Care in Injured Older Adults”. *Ann Emerg Med*. 2014; 65(1): 1-9.  **Study design**  Prognostic cross-sectional study  (Ohio Trauma Registry)  **Aim of the study**  “to evaluate the sensitivity of the Ohio geriatric trauma triage criteria compared with the adult triage criteria in identifying need for trauma center care among injured older adults.”  **Setting**  USA, 2006-2011 | **Inclusion criteria**   - injured patients - age ≥16 y - initially transported from the scene by emergency medical service personnel   **Exclusion criteria**   - absent data for ISS   **Patient characteristics**  Age [y], mean ± SD  Adults: 42 ± 16 Geriatrics: 82 ± 7  Male gender, n (%)  Adults: 45589 (66.9) Geriatrics: 10407 (31.2)  Blunt mechanism of injury, n (%)  Adults: 58449 (86) Geriatrics: 32965 (99)  Penetrating mechanism of injury, n (%)  Adults: 8445 (12)  Geriatrics: 252 (0.8)  Burns mechanism of injury, n (%)  Adults: 952 (1.4) Geriatrics: 139 (0.4)  Asphyxial mechanism of injury, n (%)  Adults: 352 (0.5) Geriatrics: 23 (0.1)  ISS, mean ± SD  Adults: 12 ± 11 Geriatrics: 8 ± 7 | **Participants**  N=101,577 patients overall;  N=33,379 geriatrics (age ≥70 y);  N=68,198 adults (age 16-69 y)  **(Potential) activation criteria**  1: Geriatric triage criteria:   - Systolic blood pressure <100 mm Hg, or absent radial pulse with carotid pulse present - GCS score ≤14 in trauma patient with a known or suspected traumatic brain injury - Fracture of 1 proximal long bone sustained from motor vehicle crash - Injury sustained in 2 or more body regions - Pedestrian struck by motor vehicle - Fall from any height, including standing falls, with evidence of a traumatic brain injury   2: Adult triage criteria   - Systolic blood pressure <90 mm Hg, or absent radial pulse with carotid pulse present - GCS score ≤13 - Fractures of 2 or more proximal long bones | **Prediction of ISS >15**  Sensitivity of geriatric criteria, % (95% CI)  Geriatrics: 93 (92 to 93) Adults: 94 (94 to 95)  Sensitivity of adult criteria, % (95% CI)  Geriatrics: 61 (60 to 62) Adults: 87 (86 to 87)  Specificity of geriatric criteria, % (95% CI)  Geriatrics: 49 (48 to 49) Adults: 35 (35 to 35)  Specificity of adult criteria, % (95% CI)  Geriatrics: 61 (61 to 62) Adults: 44 (44 to 45)  AUC of geriatric criteria, % (95% CI)  Geriatrics: 0.71 Adults: 0.65  AUC of adult criteria, % (95% CI)  Geriatrics: 0.61 Adults: 0.65  Difference in sensitivity between criteria, % (95% CI)  Geriatrics: 32 (30 to 33) Adults: 8 (7 to 8)  Difference in specificity between criteria, % (95% CI)  Geriatrics: -12 (-12 to -13) Adults: -9 (-9 to -9)  **Prediction of Operating Room Visit within 48h of injury**  Sensitivity of geriatric criteria, % (95% CI)  Geriatrics: 47 (46 to 49) Adults: 73 (72 to 73)  Sensitivity of adult criteria, % (95% CI)  Geriatrics: 35 (34 to 37) Adults: 65 (64 to 65)  Specificity of geriatric criteria, % (95% CI)  Geriatrics: 42 (41 to 42) Adults: 27 (26 to 27)  Specificity of adult criteria, % (95% CI)  Geriatrics: 57 (56 to 58) Adults: 36 (35 to 36)  AUC of geriatric criteria h, % (95% CI)  Geriatrics: 0.44 Adults: 0.5  AUC of adult criteria, % (95% CI)  Geriatrics: 0.46 Adults: 0.5  Difference in sensitivity between criteria, % (95% CI)  Geriatrics: 12 (11 to 13) Adults: 8 (8 to 8)  Difference in specificity between criteria, % (95% CI)  Geriatrics: -16 (-15 to -16) Adults: -9 (-9 to -9)  **Prediction of ICU stay of 1 day or longer during the hospitalization**  Sensitivity of geriatric criteria, % (95% CI)  Geriatrics: 81 (80 to 82) Adults: 91 (90 to 91)  Sensitivity of adult criteria, % (95% CI)  Geriatrics: 56 (55 to 57) Adults: 82 (82 to 83)  Specificity of geriatric criteria, % (95% CI)  Geriatrics: 48 (47 to 48) Adults: 34 (33 to 34)  Specificity of adult criteria, % (95% CI)  Geriatrics: 61 (60 to 62) Adults: 42 (42 to 43)  AUC of geriatric criteria, % (95% CI)  Geriatrics: 0.64 Adults: 0.62  AUC of adult criteria, % (95% CI)  Geriatrics: 0.58 Adults: 0.62  Difference in sensitivity between criteria, % (95% CI)  Geriatrics: 25 (24 to 26) Adults: 8 (8 to 9)  Difference in specificity between criteria, % (95% CI)  Geriatrics: -13 (-13 to -13) Adults: -9 (-9 to -9)  **Prediction of inhospital mortality**  Sensitivity of geriatric criteria, % (95% CI)  Geriatrics: 90 (89 to 91)  Adults: 99 (99 to 100)  Sensitivity of adult criteria, % (95% CI)  Geriatrics: 74 (72 to 76) Adults: 98 (97 to 98)  Specificity of geriatric criteria, % (95% CI)  Geriatrics: 45 (45 to 46) Adults: 30 (29 to 30)  Specificity of adult criteria, % (95% CI)  Geriatrics: 60 (60 to 61) Adults: 39 (39 to 39)  AUC of geriatric criteria, % (95% CI)  Geriatrics: 0.68 Adults: 0.64  AUC of adult criteria, % (95% CI)  Geriatrics: 0.67 Adults: 0.68  Difference in sensitivity between criteria, % (95% CI)  Geriatrics: 16 (14 to 17) Adults: 2 (0 to 2)  Difference in specificity between criteria, % (95% CI)  Geriatrics: -15 (-15 to -15) Adults: -9 (-9 to -10) | **Level of evidence**  2b  **Risk of bias**  no tool available for prognostic studies  **Authors’ conclusion**  “we demonstrated that application of Ohio’s geriatric trauma triage guidelines to the older adult population would result in improved sensitivity, with acceptable decreases in specificity for older adults. We showed that current standard adult triage guidelines provide poor sensitivity in identifying older adults with moderate to severe injury who need trauma center care. In addition, we found that using the geriatric trauma triage guidelines in younger adults provides minimal appreciable increase in sensitivity, but substantial decreases in specificity”  **Reviewers’ conclusion**  Four different common outcome measures were used to assess the need for trauma center care. The results need to be interpreted in the context of a study population with high severity of injury which might affect the test characteristics. Since the trauma registry was not designed to capture the presence of geriatric criteria, a coding scheme had to be used to identify patients who meet either of these criteria. Multiple imputation methods were used to account for a high amount of missing data. Sensitivity analyses for complete data and a restriction to 2009-2011 patients revealed comparable results. |
| **Kalkwarf (2021)**  “Prehospital ABC score accurately forecasts patients who will require immediate resource utilization”. South Med J 2021; 114($): 193-198.  **Study design**  Prognostic cross-sectional study  **Aim of the study**  “to evaluate the ability of the Prehospital ABC Score to predict blood transfusions and the need for emergent laparotomy”  **Setting**  USA, 2010 | **Inclusion criteria**   - Trauma patients - who arrived via the institution’s aeromedical transport service - underwent in-flight pFAST by a Life Flight nurse or paramedic   **Exclusion criteria**  n.r.  **Characteristics**  Age [y], median (IQR)  Prehospital ABC+: 30 (21-48) Prehospital ABC-: 40 (27-52), p=0.081  Male gender, %  Prehospital ABC+: 61 Prehospital ABC-:72, p=0.257  ISS, median (IQR)  Prehospital ABC+: 24 (14-30) Prehospital ABC-: 15 (9 to 22), p=0.023 | **Participants**  N=291 patients  **(Potential) activation criteria**  Prehospital ABC* + [positive] (N=25) Prehospital ABC* - [negative] (N=266)  *≥2 of the following present during aeromedical transport:   - Penetrating trauma - Heart rate >120 bpm - SBP <90 mmHg - Positive abdominal pFAST | Sensitivity, %  Emergent laparotomy*: 46 Massive transfusion**: 33 Substantial bleeding: 41  Specificity, %  Emergent laparotomy*: 96 Massive transfusion**: 93 Substantial bleeding: 94  Positive predictive value, %  Emergent laparotomy*: 48 Massive transfusion**: 28 Substantial bleeding: 34  Negative predictive value *, %  Emergent laparotomy*: 95 Massive transfusion**: 94 Substantial bleeding: 96  AUROC  Emergent laparotomy*: 0.836 Massive transfusion**: 0.771 Substantial bleeding: 0.849  RBC [units] in ED, median (IQR)  Prehospital ABC+: 0 (0 to 1) Prehospital ABC-: 0 (0 to 0), p<0.001  ED plasma [units], median (IQR)  Prehospital ABC+: 0 (0 to 1) Prehospital ABC-: 0 (0 to 0), p=0.002  RBC [units] after 0-3 h, median (IQR)  Prehospital ABC+: 0 (0 to 3) Prehospital ABC-: 0 (0 to 0), p<0.001  Plasma after 0-3 h, median (IQR)  Prehospital ABC+: 0 (0 to 3) Prehospital ABC-: 0 (0 to 0), p<0.001  In-hospital mortality  Prehospital ABC+: 22 Prehospital ABC-: 2, p<0.001  Cave: It is unclear whether data for mortality were reported as proportions or absolute numbers  ***** defined as taken to the OR within 2 h of admission  ** defined as transfusion of ≥10 u of packed RBCs in the first 24h after admission | **Level of evidence**  3b↓  **Risk of bias**  no tool available for prognostic studies  **Authors’ conclusions**  “The prehospital ABC score effectively predicts in-hospital resource utilization. It […] is helpful to improve trauma team activation, mobilize blood products, and prepare the operating room.”  **Reviewers’ conclusion**  The study was downgraded, because it was a posthoc analysis of a single-center study. The high false positive rate resulted in a relatively low sensitivity and positive predictive value. Only 25% of transported patients received a complete examination. Reasons for non-performance are unknown. |
| **Lehmann (2009)**  “A Simplified Set of Trauma Triage Criteria to Safely Reduce Overtriage”. *Arch Surg* 2009; 144(9): 853-858.  **Study design**  Prognostic cross-sectional study  **Aim of the study**  “To prospectively evaluate the performance of our institution’s current triage system compared with a simplified system using only 4 highly predictive variables”  **Setting**  USA, 2007-2008 | **Inclusion criteria**   - trauma patients - age >15 y   **Exclusion criteria**  n.r.  **Characteristics (overall)**  Age [y], mean  40  Blunt injury mechanism, %  95 | **Participants**  N=244 patients  **(Potential) activation criteria**  1: institution’s current triage system including 3 steps:  Step 1) Assess vital signs and level of consciousness:   - Systolic blood pressure <90 mm Hg - Heart rate >120 beats/min - For pediatric patients (aged <15 years), use blood pressure <90 mm Hg or cap refill >2 seconds - For pediatric patients (aged <15 years), use heart rate <60 or >120 beats/min - Any of the above vital signs associated with signs and symptoms of shock AND/OR - Respiratory rate <10 or >29 breaths/min associated with evidence of distress AND/OR - Altered mental status (altered neuron examination results ranging from completely unconscious to responding to painful stimuli only, or a verbal response that is confused, or an abnormal motor response)   Step 2) Assess anatomy of injury   - Penetrating injury of head, neck, torso, groin OR - Combination of burns >20% or involving face or airway OR - Amputation above wrist or ankle OR - Spinal cord injury OR - Flail chest OR - ≥2 Obvious proximal long bone fractures   Step 3) Assess biomechanics of injury and other risk factors   - Death of same-car occupant OR - Ejection of patient from enclosed vehicle OR - Falls >20 feet OR - Pedestrian hit at >20 mph or thrown 15 feet - High energy transfer situation (rollover, motorcycle/all-terrain vehicle/bicycle accident, extrication time >20 minutes - Extremes of age, <15 or >60 years - Hostile environment (extremes of heat or cold) - Medical illness (chronic obstructive pulmonary disease, congestive heart failure, renal failure etc.) - Second or third trimester of pregnancy - Gut feeling of medic   2: simplified triage protocol using 4 variables   - hypotension [<100 mm Hg in the field or ED] - mental status [GCS <14] - altered respirations - penetrating truncal wound | Accuracy (%) fraction of patients correctly classified as severely injured (need for emergency intervention*)  Current system: 21 (overall)  Simplified system: 58 (overall), p<0.05  step 1) of the current system: 36  step 2) of the current system: 31  step 3) of the current system: 7 p<0.05 for step 1 or 2 vs step 3)  *requiring an urgent procedure in the ED (intubation, tube thoracostomy, blood transfusion, resuscitative thoracotomy) and/or emergent transfer to the OR (laparotomy, craniotomy, vascular procedure, angioembolization) for a lifesaving procedure  Negative predictive value [Sensitivity according to the authors]**, %  Current system: 99.6 Simplified system: 96  **The authors seem to have confuse sensitivity and negative predictive value. This is the negative predictive value, according to the description of their calculations.  Positive predictive value [Specificity according to the authors]***, %  Current system: 21  Simplified system: 58*** The authors seem to have confuse specificity and positive predictive value. This is the positive predictive value, according to the description of their calculations. Univariate analysis for association between individual criteria and need for urgent intervention  Significant association for all step 1 criteria of the current system and all criteria of the simplified system (p<0.05)  Penetrating truncal injury was the only criterion of level 2 and level 3 criteria with a significant association | **Level of evidence**  2b  **Risk of bias**  no tool available for prognostic studies  **Authors’ conclusion**  „Using a simplified triage system can safely reduce the rate of overtriage. This could conserve resources, reduce mistriage from misunderstood guidelines, and improve specificity by including only those variables with high predictive value.“  **Reviewers’ conclusion**  This was a prospective single center study. Only few inclusion criteria and patient characteristics were reported. Results for regression analysis were only reported narratively without reporting any data. Missing information on the study population should also be kept in mind when interpreting the results. Negative predictive value [sensitivity according to the authors] and positive predictive value [specificity according to the authors] were extracted instead of over- and undertriage rates, because it was not possible to judge the appropriateness of the calculation technique due to missing information. |
| **Lin (2012)**  “Do pre-hospital trauma alert criteria predict the severity of injury and a need for an emergent surgical intervention?”. *Injury* 2012; 43(9): 1381-5.  **Study design**  Prognostic cross-sectional study  **Aim of the study**  “to evaluate which pre-hospital parameters identify major trauma victims with an emphasis on a need for emergent surgical procedures”  **Setting**  USA, 2007 | **Inclusion criteria**   - patients admitted to a level one trauma center   **Exclusion criteria**   - age <15 y - thermal, chemical and electrical injuries - patients experiencing a cardiac arrest before any surgical procedure - transferals from another hospital - adequate prehospital data could not be obtained   **Characteristics (overall)**  Male gender, %  81.26  Age [y], mean ± SD  38.25 ± 18.43  Injury mechanism, %  Blunt: 69.38 Penetrating: 30.62  ISS, mean ± SD  14.32 ± 13.71 | **Participants**  N=601 patients  **(Potential) activation criteria**  Category 1 (at least 1 criterion has to be fulfilled):   - Airway: Active airway assistance beyond supplemental O_2_ (N=40) - Consciousness: BMR <5, or paralysis, or suspicion of spinal cord injury, or loss of sensation, or GCS score ≤12 (N=128) - Circulation: No radial pulse and sustained heart rate ≥120 bpm, or SBP ≤90 mmHg (N=63) - Fracture: 2 or more long bone fractures (humerus, radius, ulna, femur, tibia, fibula) (N=37) - Cutaneous: Deep penetrating injury to head, neck & torso, Amputation at or proximal to wrist or ankle (N=139) - Other: High index of suspicion (N=222) - Two or more category 1 criteria fulfilled N=81   Category 2 (at least 2 criteria have to be fulfilled; N=44 overall):   - Age: >55 years old - Airway: Respiratory rate ≥30/min - Consciousness: BMR =5 Best motor response of the GCS - Circulation: Sustained heart rate = 120 bpm - Fracture: Any long bone fracture sustained in a motor vehicle collision or fall ≥10 feet - Cutaneous: Major degloving injury, or major flap avulsion >5 inches, or gunshot wound to the extremities - Mechanism of injury: Ejection from a closed motor vehicle, or steering wheel deformity | Obvious overtriage* n/N (%)  Category 1 airway: 0/40 (0) Category 1 consciousness: 16/128 (12.5) Category 1 circulation: 13/63 (20.6) Category 1 fracture: 3/37 (8.1) Category 1 cutaneous: 26/139 (18.7) Category 1 suspicion: 96/222 (43.2) Category 1 two or more criteria: 2/81 (2.5) Category 2 two or more criteria: 21/44 (47.7)  *With N=patients meeting the activation criterion and with a hospital stay <24h and N=all patients meeting the activation criterion  overtriage**, n/N (%)  Category 1 airway: 6/40 (15) Category 1 consciousness: 38/128 (29.7) Category 1 circulation: 20/63 (31.7) Category 1 fracture: 11/37 (29.7) Category 1 cutaneous: 73/139 (52.5) Category 1 suspicion: 175/222 (78.8) Category 1 two or more criteria: 13/81 (16) Category 2 two or more criteria: 32/44 (72.7)  **with n=patients meeting the activation criterion, but not meeting the following criteria: ISS 16 or greater, need an emergent surgery, need ICU care and N=all patients meeting the activation criterion  Prediction of major trauma***, RR (95% CI)  Category 1 airway: 9.54 (3.94–23.12) Category 1 consciousness: 3.00 (1.98–4.53) Category 1 circulation: 4.60 (2.67–7.94) Category 1 fracture: 3.94 (1.92–8.11) Category 1 cutaneous: not significant Category 1 suspicion: not significant Category 1 two or more criteria: 10.31 (5.54–19.18) Category 2 two or more criteria: not significant  ***Patients were classified as major trauma victims when their calculated ISS was 16 or greater, when they needed an emergent surgery and when they needed ICU care.  Prediction of ISS ≥25, RR (95% CI)  Category 1 airway: 11.02 (5.42–22.43) Category 1 consciousness: 6.97 (4.49–10.81) Category 1 circulation: 5.23 (3.12-8.75) Category 1 fracture: 2.35 (1.18-4.69) Category 1 cutaneous: not significant Category 1 suspicion: not significant Category 1 two or more criteria: 13.81 (7.98-23.91) Category 2 two or more criteria: not significant  Prediction of emergent operation, RR (95% CI)  Category 1 airway: 6.11 (2.73–13.71) Category 1 consciousness: 4.43 (2.28–8.58) Category 1 circulation: 11.69 (5.85–23.36) Category 1 fracture: 3.01 (1.18–7.71) Category 1 cutaneous: 3.92 (2.03–7.58) Category 1 suspicion: / Category 1 two or more criteria: 12.49 (6.24–24.99) Category 2 two or more criteria: / | **Level of evidence**  2b  **Risk of bias**  no tool available for prognostic studies  **Authors’ conclusion**  “Overall, the set of trauma alert criteria system can be further simplified and enable better utilisation of resources.”  **Reviewers’ conclusion**  This is a prospective single center study. The study reports results for overtriage and relative risks for the prediction of several aspects of major trauma. Undertriage was not addressed within this study. |
| **Matsushima (2016)**  “Should we still use motor vehicle intrusion as a sole triage criterion for the use of trauma center resources?”. *Injury* 2016; 47(1): 235-8.  **Study design**  Prognostic cross-sectional study  (Los Angeles County Trauma and Emergency Medicine Information System Trauma database)  **Aim of the study**  “to assess the validity of MVI [motor vehicle intrusion] with no other criterion in field triage following motor vehicle collision”  **Setting**  USA, 2002-2012 | **Inclusion criteria**   - patients involved in a motor vehicle accident - with associated motor vehicle intrusion   **Exclusion criteria**   - other trauma center triage criteria than motor vehicle intrusion   **Characteristics**  Male gender, n (%)  ≤18 y: 271 (55.0) 19-64y: 1825 (56.7) ≥65: 163 (56.6), p=0.76  Field SBP, median (IQR)  ≤18 y: 126 (24) 19-64y: 133 (27) ≥65y: 150 (35), p<0.001  Field heart rate, median (IQR)  ≤18 y: 100 (20) 19-64y: 96 (23) ≥65y: 90 (20), p<0.001  GCS <9 in Emergency Room, n (%)  **≤**18 y: 0 (0) 19-64y: 4 (0.1) ≥65y: 0 (0), p=1.00  Shoulder belt, n (%)  **≤**18 y: 363 (73.6) 19-64y: 2640 (82.1) ≥65y:251 (87.2), p<0.001  Airbag not deployed, n (%) ≤18 y: 56 (11.4) 19-64y: 224 (7) ≥65y: 20 (6.9), p=0.002 | **Participants**  N=3998 patients overall; N=n.r. patients ≤18y old; N=n.r. patients 19-64y old N=n.r. patients ≥65y old  **(Potential) activation criteria**  Motor Vehicle Intrusion and one of the following:   - Age ≥65y (N=288) - Male gender (N=2259) - No airbag deployment (N=300) - Seat belt (N=3254) - HR>100 bpm (N=1175) - SBP<110 mmHg (N=251)   **Variables included in logistic regression**  n.r. | **Adjusted outcomes**  Logistic regression model for need of trauma center resource*, OR (95% CI)  Age ≥65y: 3.36 (2.57 to 4.40), p<0.001  Male gender: 1.23 (1.02 to 1.48), p=0.03  No airbag deployment: 1.13 (0.94 to 1.37), p=0.20  Seat belt: 0.84 (0.66 to 1.06), p=0.15  HR >100 bpm: 1.37 (1.13 to 1.66), p=0.001  SBP <110 mmHg: 2.41 (1.78 to 3.27), p<0.001  **Unadjusted outcomes**  In-hospital mortality, n (%)  <18y: 0 (0) 19-64y: 10 (0.3) ≥65y: 9 (3.1), p<0.001  Intubation in Emergency room, n (%)  <18y: 9 (1.8) 19-64y: 79 (2.5)  ≥65y: 8 (2.8), p=0.63  Discharge from Emergency room, n (%)  <18 y: 284 (57.6) 19-64y: 1630 (50.7) ≥65: 86 (29.9), p<0.001  ISS>15, n (%)  <18 y: 46 (9.5) 19-64y: 278 (8.7) ≥65: 58 (20.2), p<0.001  LOS, median (IQR)  <18 y: 2 (1)  19-64y: 2(2) ≥65: 3 (5), p<0.001  ICU admission, n (%)  <18 y: 44 (8.9) 19-64y: 353 (11) ≥65: 89 (30.9), p<0.001  ICU LOS, median (IQR)  <18 y: 2.5 (2) 19-64y: 3 (2) ≥65: 3 (5), p=0.008  Need for trauma center resource*  <18 y: 53 (10.8) 19-64y: 435 (13.5) ≥65: 92 (31.8), p<0.001  *defined as: 1) intubation in the ER, 2) non-orthopedic surgical procedures (thoracic, abdominal, vascular, neurosurgical, obstetrics/gynecological, and neurosurgical), 3) ICU admission, and 4) in-hospital mortality | **Level of evidence**  2b  **Risk of bias**  no tool available for prognostic studies  **Authors’ conclusion**  “Overall, MVI as a sole field triage criterion results in excessive overtriage. However, patients with additional risk factors including age, gender, or field vital signs might still need to be transported to the designated trauma centers”  **Reviewers’ conclusion**  With regard to the study’s aim, there seem to be no major limitations for adjusted outcomes. However, variables included in logistic regression were not reported. When interpreting unadjusted results, one should keep in mind that population characteristics varied significantly between age groups and that the youngest age group (≤18y) probably includes patients below the age of 14y. |
| **Shawhan (2015)**  “A simplified trauma triage system safely reduces overtriage and improves provider satisfaction: a prospective study”. *Am J Surg* 2015; 209(5): 856-62.  **Study design**  Prognostic cross-sectional study  **Aim of the study**  “to prospectively analyze the safety, efficacy, and surgeon satisfaction with the newly introduced triage system.”  **Setting**  USA, 2010-2013 | **Inclusion criteria**   - Trauma patients who presented to the trauma center - Age >16 y   **Exclusion criteria**  n.r.  **Characteristics***  Age [y], mean ± SD  Level 1: 37.1 ± 18.2  Level 2: 40.4 6 21.8  Male gender, n (%)  Level 1: 61 (68)  Level 2: 86 (58%)  Blunt mechanism, n (%)  Level 1: 72 (81)  Level 2: 134 (92)  Field GCS, mean ± SD  Level 1: 11.1 ± 4.5  Level 2: 14.4 ± 1.2  ISS, mean ± SD  Level 1: 13.7 ± 12.2  Level 2: 5.7 ± 6.9  Field heart rate, mean ± SD  Level 1: 95.3 ± 28.0 Level 2: 98.1 ± 18.6  Field SBP, mean ± SD  Level 1: 131.0 ± 34.9  Level 2: 135 ± 28.3*not reported for Level 3 | **Participants**  N=460 patients  **Activation criteria**  Level 1 (N=89):   - Hypotension (SBP ≤90) or age appropriate hypotension for pediatrics - GCS <13 currently - Penetrating wound to neck, chest, or abdomen - Altered respirations or intubated in field - Proximal extremity amputation - Multiple severely injured patients incoming   Level 2 (N=146):   - GCS 13-14 - Pulse >120 (or age appropriate tachycardia for pediatrics) - Mangled extremity or distal amputations - Age >65 + mechanism (excludes ground level falls) - Neurologic deficit (paralysis, suspected spinal cord injury) - Burns >20% BSA or inhalation - Multiple long bone fractures or mangled extremity - Flail chest - Peritonitis on abdominal exam - Pregnancy   Level 3/trauma consultation (N=225):   - All other traumatic mechanisms - GCS 15 and normal vital signs | **Accuracy, n/N (%)**  fraction of patients correctly classified as severely injured (patients requiring ICU admission), with N=all patients meeting the activation level  Level 1: 56/89 (63)  Level 2: 26/146 (18) Level 3: n.r.  fraction of patients correctly classified as severely injured (requiring urgent intervention), with N=all patients meeting the activation level  Any intervention  Level 1: 50/89 (56)  Level 2: 8/146 (5) Level 3: 7/225 (3)  Intubation  Level 1: 28/89 (31) Level 2: 1/146 (0.7) Level 3: 4/225 (1.8)  Surgical airway  Level 1: 1/89 (1) Level 2: 0/146 (0) Level 3: 0/225 (0)  Chest tube  Level 1: 14/89 (16) Level 2: 2/146 (1) Level 3: 1/225 (0.4)  Central line  Level 1: 11/89 (12) Level 2: 2/146 (1) Level 3: 0/225 (0)  Blood products  Level 1: 12/89 (13) Level 2: 5/146 (3) Level 3: 1/225 (0.4)  Cardiopulmonary resuscitation  Level 1: 5/89 (6) Level 2: 0/146 (0) Level 3: 0/225 (0)  Urgent surgery  Level 1: 12/89 (13) Level 2: 1/146 (0.7) Level 3: 2/225 (0.9) | **Level of evidence**  2b  **Risk of bias**  no tool available for prognostic studies  **Authors’ conclusion**  n.r. for comparison of interest  **Reviewers’ conclusion**  This was a prospective single center study. The low number of patients for some outcomes to be predicted should be kept in mind when interpreting the results for individual TTA criteria. Data for the predictive value of level 3 activation criteria for ICU admission are missing. |
| **Singh (2014)**  “Correlation of Shock Index and Modified Shock Index with the Outcome of Adult Trauma Patients: A Prospective Study of 9860 Patients”. *N Am J Med* 2014; 6(9): 450-2.  **Study design**  Prognostic cross-sectional study  **Aim of the study**  **“**to evaluate the predictive value of shock index (SI) and modified shock index (MSI) for hospital mortality among adult trauma patients.”  **Setting**  India, 2013 | **Inclusion criteria**   - Adult patients - With trauma - Presenting to the Emergency Room   **Exclusion criteria**   - Referrals from another hospital after the first aid - death within the first six hours of arrival - patients <18 y - incomplete data   **Characteristics (overall)**  Age [y], mean ± SD  39 ± 11  Male gender, %  63  HR [beats/minute], mean ± SD  103 ± 17  SBP [mmHg], mean ± SD  126 ± 19  DBP [mmHg], mean ± SD  69 ± 15  SI, mean ± SD  0.69 ± 0.23  MSI, mean ± SD  1.08 ± 0.20 | **Participants**  N=9860 patients  **(Potential) activation criteria**   - Heart rate >120 - SBP <90 mmHg - Diastolic blood pressure <60 mmHg - Shock index (heart rate/SBP)<0.5 - Shock index (heart rate/SBP)>0.9 - Modified shock index (heart rate/mean arterial pressure) <0.7 - Modified shock index (heart rate/mean arterial pressure) >1.3   **Criteria included in logistic regression**   - Heart rate - SBP - Diastolic blood pressure - Shock index - Modified shock index | Prediction of mortality based on logistic regression, OR (95% CI)  HR >120: 2.5 (1.7 to 3.3)  SBP <90: 2.6 (1.9 to 3.4)  DBP <60: 1.9 (1.4 to 2.3)  SI <0.5: 1.3 (0.8 to 1.6)  SI >0.9: 1.1 (0.7 to 1.7)  MSI <0.7: 3.5 (2.1 to 6.9)  MSI >1.3: 4.5 (2.9 to 6.6) | **Level of evidence**  2b  **Risk of bias**  no tool available for prognostic studies  **Authors’ conclusion**  “MSI, as a potential marker for predicting the mortality rate and is significantly better than HR, SBP, diastolic blood pressure, and SI alone. Thus, MSI emerges as a better and improved predictor for prediction of hospital mortality in adult trauma patients in the emergency room”  **Reviewers’ conclusion**  This was a prospective single center study. When interpreting the results, one should keep in mind that high number of patients (14.2%) where excluded due to missing data. Moreover, 249 patients were excluded, because of death within six hours of hospital arrival. Clinical data (eg, heart rate, SBP) were collected at the time of admission and then hourly. Furthermore, MSI and SI were calculated at six hours. This might limit transferability of results to pre-clinical settings. Results on the predefined endpoints ICU stay and hospital stay are missing. |
| **St. John (2018)**  “Prehospital Lactate Predicts Need for Resuscitative Care in Non-hypotensive Trauma Patients”. *West J Med* 2018; 19(2): 224.  **Study design**  Secondary analysis of a prognostic cross-sectional study  **Aim of the study**  “to determine the test characteristics of prehospital lactate levels for predicting need for resuscitative care among a broad population of normotensive trauma patients being transported by ground ALS units”  **Setting**  North America, 2011-2012 | **Inclusion criteria**   - patients who received intravenous access - and were transported to a Level I trauma center - valid prehospital lactate measurement   **Exclusion criteria**   - prehospital systolic blood pressure ≤100 mmHg - age <15 years - obvious isolated, penetrating head trauma - drowning - asphyxia caused by hanging - burns >20% body surface area - known prisoner status   **Characteristics (overall)**  Age [y], median (IQR)  35.5 (25-51)  Male, n (%)  228 (72.6)  Mechanism of injury, n (%)  Blunt 260 (82.8 Penetrating 54 (17.2)  ISS, median (IQR)  9 (5-19) | **Participants**  N=314 patients  **(Potential) activation criteria**   - Prehospital lactate - Shock index (heart rate/SBP)   **Variables included in multivariable logistic regression**  n.r. | **Adjusted outcomes**  Logistic regression analysis for need for resuscitative care*, OR (95% CI)  Prehospital lactate**:  1 mmol/L difference in prehospital lactate within the range of <2.5: 1.29 (0.40 – 4.12), p=0.666  1 mmol/L difference in prehospital lactate within the range of 2.5-4.0: 2.27 (1.10 – 4.68), p=0.027  1 mmol/L difference in prehospital lactate within the range of ≥4.0: 1.26 (1.05 – 1.50), p=0.011  **Unadjusted outcomes**  AUROC for need for resuscitative care*, % (95% CI)  Prehospital lactate 0.716 ([0.632 – 0.800)  Shock index 0.631 (0.537 – 0.724), p=0.125  Sensitivity for need for resuscitative care*, % (95% CI)  Prehospital lactate level ≥2.5 mmol/L: 74.6  Prehospital lactate level ≥3.0 mmol/L: 70.9  Shock index ≥0.9: 30.8  Either prehospital lactate level ≥2.5 mmol/L or shock index ≥0.9: 77.6  Specificity for need for resuscitative care*, % (95% CI)  Prehospital lactate level ≥2.5 mmol/L: 53.4  Prehospital lactate level ≥3.0 mmol/L: 66.9  Shock index ≥0.9: 89.9  Either prehospital lactate level ≥2.5 mmol/L or shock index ≥0.9: 49.8  *need for resuscitative care defined as either   - death in the ED - disposition to operating room or interventional radiology within six hours - receipt of five units of blood within six hours of ED arrival.   **a multivariate logistic regression was performed that included need for resuscitative care as its outcome and a linear spline of lactate with knots at 2.5 and 4 mmol/L as the predictor of interest. | **Level of evidence**  3b↓  **Risk of bias**  no tool available for prognostic studies  **Authors’ conclusion**  “In conjunction with previous studies on prehospital lactate in trauma patients, these findings suggest that prehospital lactate could improve overall triage for ALS patients, and we suggest that it should be investigated prospectively as a rapid test in the field to identify occult shock”  **Reviewers’ conclusion**  The study was downgraded, because it was a secondary analysis. The authors stated that the impact of inclusion of prehospital lactate into any triage protocol would need to be investigated prospectively before it could be recommended for implementation. |
| **Tignanelli (2018)**  “Noncompliance with American College of Surgeons Committee on Trauma recommended criteria for full trauma team activation is associated with undertriage deaths”. *J Trauma Acute Care Surg* 2018; 84(2): 287-294.  **Study design**  Prognostic cross-sectional study and comparative registry study  (Michigan Trauma Quality Improvement Program)  **Aim of the study**  “we examined the compliance rate of ACS-COT–verified Level I and II trauma centers in Michigan with the ACS-6 triage criteria. We evaluated the association of these criteria with trauma patient mortality and rates of emergent intervention.”  **Setting**  USA, 2014-2016 | **Inclusion criteria**   - Age ≥16 y - ISS ≥5 - At least one valid trauma International Classification of Diseases, 9th Revision, Clinical Modification code in the range of 800 to 959.9 - Primary mechanism of injury classified as either blunt or penetrating - ED discharge disposition and hospital discharge disposition known   **Exclusion criteria**   - Patients directly admitted - Missing data - no signs of life at initial evaluation (ED SBP, 0; pulse, 0; GCS score, 3) - late effects (905–909.9) - superficial injuries (910–924.9) - foreign bodies (930–930.9)   **Characteristics**  Age 18-25 [y], %  1: 14 2: 21 3: 40 4: 22 5: 24 6: 10  Age 26-45 [y], %  1: 22 2: 29 3: 44  4: 29 5: 31 6: 16  Age 46-65 [y], %  1: 32 2: 28 3: 13 4: 27 5: 25 6: 25  Age 66-75 [y], %  1: 12 2: 10 3: 2 4: 9 5: 8 6: 14  Age >75 [y], %  1: 20 2: 13 3: 1  4: 13 5: 12  6: 35  Male gender, %  1: 64 2: 74 3: 88 4: 73 5: 75 6: 53  GCS score 14-15, %  1: 66 2: 19 3: 77 4: 0 5: 45 6: 88  GCS score 9-13, %  1: 9 2: 15 3: 5 4: 0 5: 10 6: 3  GCS score 3-8, %  1: 21 2: 61 3: 15 4: 100 5: 41 6: 0  ISS 5-15, %  1: 52 2: 27 3: 62 4: 24 5: 45 6: 86  ISS 16-24, %  1: 21 2: 24 3: 18 4: 22 5: 21 6: 11  ISS 25-35, %  1: 17 2: 36 3: 18 4: 38 5: 26 6: 3  ISS >35, %  1: 11 2: 13 3: 2 4: 16 5: 9 6: 0.3  ED SBP≥91 [mmHg], %  1: 0 2: 84 3: 86 4: 83 5: 75 6: 97   - ED SBP 61-90 [mmHg], %   1: 83 2: 9 3: 8 4: 8 5: 18 6: 0  ED SBP≤60 [mmHg], %  1: 17 2: 3 3: 3 4: 3 5: 4 6: 0 | **Participants**  N=51,792 patients  **Prognostic part**  **(Potential) activation criteria**  1: SBP ≤90 mmHg (N=1346)  2: Intubation (N=3459)  3: Central gunshot wound (N=1931)  4: GCS score <9 (N=2475)  5: Any ACS-6 criterion including the 4 mentioned above + Transfer patients from other hospitals receiving blood to maintain vital signs & emergency physician’s discretion (N=6080)  Cave: It remains unclear, whether N=6080 includes all ACS-6 criteria or only criteria 1-4.  6: no ACS-6 criterion (N=45712)  **Intervention comparison**  N=1130 patients with full activation N=243 patients with partial activation N=379 patients with trauma consult only N=382 with no activation  “Data collected reflect the highest tier of activation status and account for activation upgrades.” | Prognostic part  Accuracy, n/N (%) fraction of correctly classified as severely injured (need for emergent intervention* and meeting the activation criterion), with N=all patients meeting the activation criterion, n/N (%)  1: 848/1346 (63)  2: 3459/3459 (100) 3: 1285/1931 (67) 4: 2271/2475 (92) 5: 4781/6080 (79) 6: 6746/45712 (15)  * defined as receiving one or more of the following: transfusion of greater than four units of blood within 4 hours of arrival, emergent central line insertion, emergent operation, emergent angiography, emergent intubation, emergent chest tube placement, or placement of a cerebral monitor.  Undertriage, n/N (%)*^*^  1: 157/1346 (12)  2: 514/3459 (15) 3: 42/1931 (2.2) 4: 274/2475 (11) 5: 728/6080 (12) 6: 5572/45712 (12)  **major trauma (ISS >15) but no full TTA (due to non-compliance with the proposed activation criteria)  Mortality in the undertriaged population, n/N (%)  1: 24/157 (15)  2: 179/514 (35) 3: 10/42 (24) 4: 130/274 (47) 5: 217/728 (30) 6: 216/5572 (4)  **Intervention comparison**  Mortality for SBP **≤**90 mmHg stratified for activation levels, n/N (%)  Full activation: 208/NR (30) Partial activation: 15/NR (6) trauma consult only: 24/NR (11) no activation: 18/NR (10)  Mortality for intubation stratified for activation levels, n/N (%)  Full activation: 915/NR (35) Partial activation: 96/ NR (19) trauma consult only: 82/NR (35) no activation: 47/NR (39)  Mortality for Central gunshot wound stratified for activation levels, n/N (%)  Full activation: 278/NR (19) Partial activation: 0/NR (0) trauma consult: 11/NR (11) no activation: 3/NR (5)  Mortality for GCS<9 stratified for activation levels, n/N (%)  Full activation: 852/NR (42) Partial activation: 54/NR (25) trauma consult: 67/NR (49) no activation: 36/NR (41)  Mortality for any ACS-6 criterion stratified for activation levels, n/N (%)  Full activation: 1057/NR (26) Partial activation: 110/NR (10) trauma consult: 119/NR (21) no activation: 72/NR (19)  Mortality for no ACS-6 criterion stratified for activation levels, n/N (%)  Full activation: 73/NR (3) Partial activation: 133/NR (1) trauma consult: 260/NR (2) no activation: 310/NR (2) | **Level of evidence**  2b  **Risk of bias**  Prognostic part:  no tool available for prognostic studies  Intervention comparison:  Selection bias: –  Performance bias: ?  Attrition bias: +  Detection bias: ?  **Authors’ conclusion**  n.r. for comparison of interest  **Reviewers’ conclusion**  Prognostic part:  Two out of 6 criteria (Transfer patients from other hospitals receiving blood to maintain vital signs & emergency physician’s discretion) proposed by the American College of Surgeons Committee on Trauma could not be identified in the available database. Therefore, it remains unclear, whether criterion 5 includes all 6 ACS-criteria in an aggregated form or only criteria 1-4. This might have an impact on the results for criterion 5 and 6. When interpreting the undertriage rate and mortality in the undertriaged population one should keep in mind that these patients did not receive full TTA due to non-compliance with the proposed activation criteria.  Intervention comparison:  There is a high risk of selection bias. The risk of bias remains unclear for this part of the study due to lack of information. |
| **Werman (2011)**  **“**Development of Statewide Geriatric Patients Trauma Triage Criteria”. Prehosp Disaster Med 2011; 26(3): 170-179.  **Study design**  Prognostic cross-sectional study  (Ohio Trauma Registry)  **Aim of the study**  “describes the development of geriatric- specific field destination criteria for the state of Ohio”  **Setting**  USA, 2003-2006 | **Inclusion criteria**  n.r.  **Exclusion criteria**  n.r.  **Characteristics**  n.r. | **Participants**  N=90,597 overall;  N=n.r. for geriatrics (>70 y);  N=n.r. for adults (16-69 y)  **(Potential) activation criteria**  1: GCS  2: SBP  3: falls associated with head, chest, abdominal or spinal injury  4: pedestrian struck  5: multiple body regions injured  6: motor vehicle collision with humerus/femur fracture | Prediction of mortality by different GCS levels % (95% CI)  GCS 13: Geriatrics: 14.4 (11.1 to 18.5) Adults: 3.9 (2.8 to 5.0)  Geriatrics with a GCS=14 vs. adults with GCS=13, RR (95% CI):  1.65 (1.14–2.40),p=0.01  🡪 “the Task Force recommended that a GCS of 14 or less for an elderly patient be used as a criterion for transport to a trauma center […] an additional qualifier was added to suggest that there must be some evidence of recent head trauma for triage to a trauma center.”  Prediction of mortality by different SBP levels, % (95% CI)  SBP 81–90 mmHg: Geriatrics: 19.2 (15.1 to 23.2) Adults: 12.0 (10.1 to 14.0)  Geriatrics with SBP 91–100 mmHg vs. adults with SBP of 81–90 mmHg, RR (95% CI)  0.98 (0.73–1.32),p=0.89  Geriatrics with SBP 101–110 mmHg vs. adults with SBP 81–90 mmHg, RR (95% CI)  0.69 (0.53–0.90),p=0.005  🡪”the Task Force proposed that elderly trauma patients with a SBP of <100 mmHg be evaluated in a trauma center.”  Prediction of mortality for falls with traumatic brain injury, OR (95% CI)  2.12 (1.88 to 2.39), p<0.001  Prediction of mortality for falls with traumatic chest injury, OR (95% CI)  1.22 (0.99 to 1.52), p=0.056  Prediction of mortality for falls with traumatic pelvic/abdominal injury, OR (95% CI)  0.98 (0.73 to 1.31), p=0.865  Prediction of mortality for falls with traumatic spinal cord injury, OR (95% CI)  1.22 (0.99 to 1.52), p=0.056  🡪“The Task Force proposed […] that geriatric trauma patients with falls and evidence of traumatic brain injury (regardless of GCS score) should be triaged to a trauma center.”  Prediction of mortality for pedestrian struck, OR (95% CI  2.39 (1.77 to 3.21), p=0.001  🡪”The Task Force proposed that geriatric pedestrians who are struck by a moving vehicle be triaged to a trauma center.”  Prediction of mortality for multiple body regions injured, OR (95% CI  1.29 (1.06 to 1.57), p=0.01  🡪”it was recommended that geriatric patients with injuries to more than one body system be included in the revised field destination guidelines and ultimately would be assessed in a trauma center.”  Prediction of mortality motor vehicle collision with humerus/femur fracture, OR (95% CI  2.41 (1.81 to 3.21), p=0.001  🡪”the presence of any proximal long-bone fracture following motor vehicle trauma would require an evaluation in a trauma center in the proposed triage scheme.” | **Level of evidence**  3b↓  **Risk of bias**  no tool available for prognostic studies  **Authors’ conclusion**  “As a result of this analysis, the Task Force recommended additional geriatric-specific criteria that are listed in Table 6. These include an increase in both the GCS score and SBP used to determine field destination for geriatric patients, as well as the addition of single long-bone fracture in motor vehicle collision and pedestrian struck as definitive destination criteria. These criteria represent changes or additions to the existing criteria for other adult trauma patients”  **Reviewers’ conclusion**  The study was downgraded, because no inclusion and exclusion and no patient characteristics were provided. The authors state that the generalizability to other settings might be limited. Only results for mortality were reported even though the authors state they found conflicting findings for the adult and geriatric populations when total length of stay, ICU length of stay, and number of complications were investigated. |
| +: low risk; –: high risk; ?: unclear risk  ACS: American College of Surgeons Committee on Trauma; adj.: adjusted; AIC: Akaike information criterion; AIS: Abbreviated Injury Scale; AUROC: area under the ROC curve; BMR: Best motor response of the GCS; bpm: beats per minute; BSA: body surface area; CI: Confidence Interval; d: days; DBP: Diastolic Blood Pressure; ED: Emergency Room; EMS: Emergency Medical Service; ER: Emergency Room; GCS: Glasgow Coma Scale; h: hours; HR: Hazard Ratio; HR: Heart Rate; ICU: Intensive Care Unit; IQR: Interquartile Range; ISS: Injury Severity Score; ITT: Intention to Treat; LOS: Length of Stay; m: months; MOI: Mechanism of Injury; MSI: Modified Shock Index; MVI: Motor Vehicle Injury; n.r.: not reported; NTTP: National Trauma Triage Protocol; OR: Odds Ratio; PR: pulse rate; RBC: Red Blood Cells; ROC: receiver operating characteristic; RPBC: Red Packed Blood Cells; RR: Relative Risk; RR: respiratory rate; SBP: Systolic Blood Pressure; SD: Standard Deviation; SEM: Standard Error of Mean; SI: Shock Index; TARN: Trauma Audit and Research Network; TTA: Trauma Team Activation; y: years; | | | | |

##### Polytrauma care: Teams / Training

| **Study: Reference, aim, design, setting** | **Participants: selection criteria, characteristics** | **N Participants; Intervention group (IG) vs. control group (CG)** | **Main outcomes** | **Assessment: LoE, risk of bias; Conclusions** |
| --- | --- | --- | --- | --- |
| **Daurka (2015)**  “A priority driven ABC approach to the emergency management of high energy pelvic trauma improves decision making in simulated patient scenarios”. Injury 2015; 46(2): 340-3.  **Study design**  RCT  **Aim of the study**  “to assess whether trainees taught this ABC initialled aide memoire gave better priority driven care in simulated patient scenarios. They were compared directly to their colleagues that underwent the same pelvic training but without reference to the ABC concept.”  **Setting**  UK, n.r. | **Inclusion criteria**   - orthopaedic trainees - belonging to the same deanery teaching group with similar levels of training and experience in pelvic trauma   **Exclusion criteria**   - Those completing a post with a strong element of pelvic trauma or previously completed a pelvic trauma training course   **Characteristics**  Year of training, mean  IG: 4.4 CG: 4.9, p=0.426  Number of high energy pelvic cases treated, mean  IG: 3  CG: 1.4, p=0.347 | **Participants**  N=20 trainees  **Study groups**  IG: pelvic training afternoon (1h presentation of the principles of pelvic trauma patient management including classification, associated injuries, early and definitive management led by two experienced pelvic and acetabular surgeons)+ 5 slides covering the ABC concept (N=11)  CG: pelvic training afternoon only (N=9) | Improved responses in assessment and management of possible coagulopathy*, %  IG: 55 CG: 0, p<0.001  Improved responses in assessment and management of possible urological pathology*, %  IG: 97 CG: 78, p=0.047  Improved responses in assessment and management of possible bowel injury/open fracture*, %  IG: 60 CG: 26, p=0.007  improved responses for resuscitation*, %  IG: 78 CG: 74, p=0.67  Improved responses for blood and blood products and in the initiation of antimicrobial therapy*, %  IG: 33 CG: 15, p=0.09  Worsened responses for CT scanning*, %  IG: 45 CG: 81, p=0.004  Number of appropriately prioritised management plans*, %  IG: 78 CG: 44, p=0.006  *assessed after 6 weeks using viva scenarios of pelvic trauma | **Level of evidence**  2b↓  **Risk of bias**  Selection bias: ?  Performance bias: +  Attrition bias: +  Detection bias: +  **Authors’ conclusion**  “This study has demonstrated that using an initialled ABC acronym as a teaching method improves both clinician’s recall and prioritisation when discussing pelvic trauma in examination scenarios”  **Reviewers’ conclusion**  This was an RCT which was downgraded because of a small sample size. The outcomes were measured after 6 weeks. The study did not account for long term outcomes. The authors do not refer to a study protocol. |
| +: low risk; –: high risk; ?: unclear risk CG: Comparison group; CT: computed tomography; h: hours; IG: Intervention group; RCT: randomised controlled trial | | | | |

# S5 Deleted Recommendations

| **Deleted recommendation** | **Reason** |
| --- | --- |
| Ensure that senior physicians required for further care are present within the next 20-30 minutes after being requested. | To date, this recommendation is not legally tenable. Therefore it was deleted, but discussed in the background text of the original guideline |
| The size of the resuscitation room should be 25-50 m2 (per patient to be treated). | The recommendation has been moved to another guideline chapter |
| The resuscitation room, the patient arrival area, the radiology department and the surgery department should be located in the same building. The helipad should be located on the hospital grounds. | The recommendation has been moved to another guideline chapter |

translated with DeepL https://www.deepl.com/
